# *In Vitro* and *In Silico* Antidiabetic and Antimicrobial Evaluation of Constituents from *Kickxia ramosissima* (*Nanorrhinum ramosissimum*)

**DOI:** 10.3389/fphar.2017.00232

**Published:** 2017-05-01

**Authors:** Adnan Amin, Emmy Tuenter, Kenn Foubert, Jamhsed Iqbal, Paul Cos, Louis Maes, Vassiliki Exarchou, Sandra Apers, Luc Pieters

**Affiliations:** ^1^Laboratory of Natural Products and Food Research and Analysis, Department of Pharmaceutical Sciences, University of AntwerpAntwerp, Belgium; ^2^Centre for Advanced Drug Research, COMSATS Institute of Information TechnologyAbbottabad, Pakistan; ^3^Laboratory of Microbiology, Parasitology and Hygiene, Faculty of Pharmaceutical, Biomedical and Veterinary Sciences, University of AntwerpAntwerp, Belgium

**Keywords:** *Kickxia ramosissima*, *Nanorrhinum ramosissimum*, Plantaginaceae, iridoids, antimicrobial activity, antiglycation activity

## Abstract

**Background and Aims:** *Kickxia ramosissima* (Wall.) Janch (or *Nanorrhinum ramosissimum* (Wall.) Betsche is a well-known medicinal plant in Pakistan that is traditionally used in diabetic and inflammatory conditions. Because little information is available on its phytochemical composition, a range of constituents were isolated and evaluated *in vitro* in assays related to the traditional use.

**Methods:** Dried whole plant material was extracted and chromatographically fractionated. Isolated constituents were evaluated *in silico* and *in vitro* in assays related to the traditional use against diabetes (inhibition of α-glucosidase activity; inhibition of advanced glycation endproducts) and in inflammatory conditions (inhibition of AAPH induced linoleic acid peroxidation, inhibition of 15-LOX, antimicrobial activity).

**Results:** Phytochemical analysis of the extracts and fractions led to isolation of **7** compounds, including the iridoids kickxiasine (being a new compound), mussaenosidic acid, mussaenoside and linarioside; the flavonoids pectolinarigenin and pectolinarin; and 4-hydroxy-benzoic acid methyl ester. The iridoids showed weak antiglycation activity. The flavonoids, however, showed interesting results as pectolinarigenin was highly active compared to pectolinarin. In the α-glucosidase inhibition assay, only weak activity was observed for the iridoids. However, the flavonoid pectolinarigenin showed good activity, followed by pectolinarin. In the 15-LOX experiment, moderate inhibition was recorded for most compounds, the iridoids mussaenosidic acid and mussaenoside being the most active. In the AAPH assay, weak or no inhibition was recorded for all compounds. The *in silico* assays for the α-glucosidase and 15-LOX assays confirmed the results of respective *in vitro* assays. Pectolinarigenin showed moderate antimicrobial activity against *Staphylococcus aureus*, *Plasmodium falciparum* K1, and *Trypanosoma cruzi*, but it was not cytotoxic on a human MRC-5 cell line.

**Conclusion:** Our findings may in part contribute to explain the traditional use of *K. ramosissima*.

## Introduction

*Kickxia* (Plantaginaceae) is a small genus of herbs geographically distributed from West-Africa to India (Khan and Aqil, 1993).

The genus *Kickxia* is comprised of 47 species worldwide (Mabberley, 1997). *Kickxia ramosissima* (Wall.) Janch. is a perennial herb with numerous filiform branches, membranous leaves, and yellow flowers (Pandya et al., 2012). It is mainly found in rocky areas with shady places (Kirtikar and Basu, 2005) and high salt contents (Mathur et al., 1980). Although *Nanorrhinum ramosissimum* (Wall.) Betsche is the accepted name of the species in the genus *Nanorrhinum* (family Plantaginaceae) (adopted in 1984) (The Plant List, 2016), common synonyms are *Linaria ramosissima* Wall.; *Linaria somalensis* Vatke; *Kickxia ramosissima* (Wall.) Janchen; *Kickxia somalensis* (Vatke) Cuf., and *Pogonorrhinum somalense* (Vatke) Betsche (Plants Jstore, 2016). Due to the fact that synonym *Kickxia ramosissima* is more commonly used in literature, it was adopted during the current project. In Pakistan it has been reported from various places including the districts Attock (Ahmad et al., 2009), Karak (Khan et al., 2011), and Sindh (Qureshi et al., 2010). *K. ramosissima* has been used in the indigenous system of treatment of the Indian subcontinent (Vardhana, 2008). In Pakistan it is known as “Wal,” “Shin beeta” or “Khunger booti.” It is used for a number of ailments, for instance as diuretic, against kidney stones (Pandya et al., 2012), fever and rheumatism (Jain et al., 2008) and during management of snake and scorpion bites (Bole and Pathak, 1988). Traditionally in the Indian subcontinent including Pakistan this species has been reported as an effective remedy for diabetes mellitus (Qureshi and Bhatti, 2008; Ahmad et al., 2009; Qureshi et al., 2010; Patel and Sachdeva, 2014).

Despite its ethnomedicinal importance only a few *Kickxia* species worldwide were chemically investigated. This has resulted in the isolation of mainly flavonoids and iridoid glycosides (Khan and Aqil, 1993; Khan et al., 2001; Ahmad et al., 2006; Al-Rehaily et al., 2006; Ferhat et al., 2010), fatty acids (Morteza-Semnani et al., 2008), and D-mannitol (Khan and Aqil, 1993). *K. ramosissima*, however, is one of the least explored species. Because of its use in diabetic conditions, isolated constituents were evaluated for inhibition of the formation of AGEs and inhibition of α-glucosidase activity. AGEs are involved in many degenerative diseases, such as diabetes and its complications, cardiovascular and neurodegenerative diseases, and the physiological process of aging. α-Glucosidase inhibitors are able to prevent the fast breakdown of sugars by competitively inhibiting α-glycosidase activity and thus controlling the blood sugar levels. This category of oral hypoglycemic agents is important in cases of post-prandial blood glucose elevation in diabetic patients. There are quite a few commercially available α-glucosidase inhibitors, but gastrointestinal side effects limit their use. It was therefore considered important to test the isolated compounds for α-glucosidase inhibition.

Because of the traditional use in inflammatory conditions and against fever, isolated constituents were also evaluated for antioxidant activity (AAPH induced linoleic acid peroxidation), inhibition of 15-LOX and antimicrobial activities. The soybean LOX assay is used as an indication of anti-inflammatory activity (Muller, 1994). LOX is a key enzyme in the inflammatory cascade, whose inhibition is correlated to the ability of the inhibitors to reduce Fe^3+^ at the active site to the catalytically inactive Fe^2+^. Anti-inflammatory activity can result from inhibition of the arachidonic acid (AA) pathway. AA is an unsaturated (C_20_) fatty acid that is produced from membrane phospholipids. The derivatives of AA are potent mediators of inflammation. It is therefore considered that by inhibition of the biosynthesis of pro-inflammatory molecules, the inflammation can be reduced or ended by using antioxidants and anti-inflammatory drugs (Grantstrom, 1984). In the AAPH assay, inhibition of AAPH-initiated linoleic acid lipid peroxidation is evaluated. *In silico* drug targets were identified for α-glucosidase and 15-LOX inhibition.

In view of its use in inflammatory conditions and against fever, also the antimicrobial activity of crude extracts and isolated constituents was investigated in a screening panel of infectious microorganisms, including a human MRC-5 cell line to assess the selectivity.

## Materials and Methods

### Solvents and Reagents

All solvents were of analytical grade and obtained from Fisher Scientific (Leicestershire, UK) and Acros Organics (Geel, Belgium). All chemicals and reagents were purchased from Acros Organics or Sigma–Aldrich (St. Louis, MO, USA). The solvents for HPLC were purchased from Fisher Scientific (Leicestershire, UK). RiOS water was prepared by reverse osmosis and water for HPLC was dispensed by a Milli-Q system, both from Millipore (Bedford, MA, USA). Water was passed through a 0.22 mm membrane filter before usage.

### Chromatography

Analytical plates for thin layer chromatography (TLC) were silica gel 60 F_254_ plates (20 cm × 20 cm) for normal phase (Merck, Darmstadt, Germany). The spraying reagent *p*-anisaldehyde was prepared by mixing 0.5 mL *p*-anisaldehyde (Sigma–Aldrich) with 10 mL glacial acetic acid, 85 mL methanol, and 5 mL sulphuric acid.

Flash column chromatography was performed on a Reveleris iES system from Grace (Columbia, MD, USA) using the Reveleris^®^ Navigator^TM^ software. The system is equipped with a binary pump with four solvent selection, an ultraviolet (UV) and evaporating light scattering detector (ELSD) and a fraction collector. The column used was a pre-packed Flash Grace Reveleris silica cartridge (80 g) with a particle size of 40 μm. The ELSD carrier solvent was isopropyl alcohol.

HPLC analysis was carried out on an Agilent^®^ 1200 series system with degasser, quaternary pump, automatic injection, thermostatic column compartment and a diode array detector (DAD) (Agilent^®^ Technologies, Santa Clara, CA, USA). A silica based Gracesmart C_18_ column (250 mm × 4.6 mm, 5 μm) (Grace Vydac, USA) and Phenomenex luna C_18_ (250 mm × 4.6 mm, 5 μm) (Phenomenex, Torrence, CA, USA) was used together with a suitable precolumn to endure the lifetime of columns.

The isolation of compounds was carried out on a semi-preparative HPLC-DAD-MS system (Waters^®^) using a Luna 5 μ (C_18_) 100A 250 mm × 10.0 mm column (Phenomenex^TM^) and Masslynx^®^ 4.1 software. The system was equipped with HPLC pump 515 (Waters^TM^ 2767), make-up pump (Waters^TM^ 511), system fluid organizer (SFO), DAD (Waters^TM^ 2998), triple quadrupole mass spectrometer (TQD-MS) and automatic fraction collector was used to isolate compounds.

### Structure Elucidation

NMR spectra were recorded on a Bruker DRX-400 instrument (Rheinstetten, Germany), operating at 400 MHz for ^1^H and at 100 MHz for ^13^C, employing a 3-mm broadband inverse (BBI) probe or a 5-mm dual ^1^H/^13^C probe using standard Bruker pulse sequences. DEPT-135, DEPT-90, and two-dimensional NMR (COSY, HSQC, and HMBC) experiments were recorded. In order to assist structure elucidation a ^13^C NMR library was used (NMR Predict version 4.8.57, Modgraph). Deuterated solvents including CDCl_3_ (99.8% D), CD_3_OD (99.8% D), D_2_O (99.9% D) were purchased from Sigma–Aldrich.

High resolution mass spectra was obtained with an Agilent^TM^ 6530 quadrupole-time-of-flight mass spectrometer (QTOF-MS) equipped with an Agilent^TM^ Jetstream source. The mass spectrometer was operated in positive and negative ion mode at 20,000 resolution. The instrument was calibrated and tuned with a tune mix (G1969-85000) and during acquisition the accuracy was monitored by using ES-TOF reference mass solution kit (G1969-85001) from Agilent^TM^. Mass Hunter^®^ (Agilent^TM^ Technologies) software was used for acquisition and processing.

The specific optical rotation was determined on a Jasco P-2000 polarimeter. The samples were dissolved in methanol and optical rotation was recorded at 589 nm with a path length of 50 mm.

UV-VIS (ultraviolet-visible light) absorbance was measured on a Genesys-10UV (Thermoscientific) spectrophotometer. Fluorescence (excitation 335 nm, emission 385 nm, and excitation 370nm, emission 440 nm) was measured on a Tecan^TM^ Infinite M200 spectrofluorometer.

### Plant Material

*Kickxia ramosissima* whole plant was collected in October 2012 from Takht-e-Nusrati, district Karak (KPK), Pakistan. Afterward the herbarium sheets of the collected whole plant were submitted for identification at the Islamabad Herbarium in the Taxonomy Department, Quaid-I-Azam University, Islamabad, Pakistan, where the voucher specimen was deposited (voucher No. 48 CJ, accession no. ISL-44586). The whole plant was dried under shade followed by powdering and sieving through a 20 mesh filter. All powdered material was stored below 20°C till further use.

### Extraction and Isolation

**FIGURE 1:**
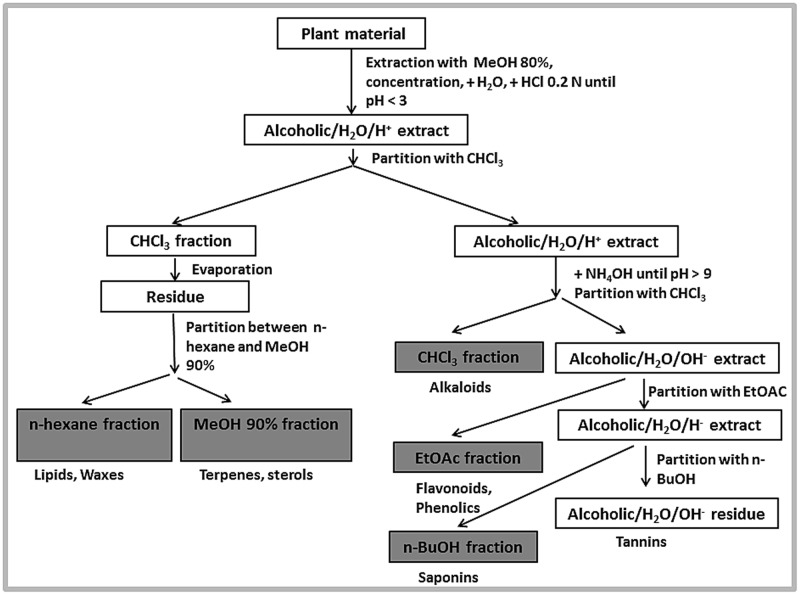
**Fractionation scheme**.

Powdered whole plant material (0.955 kg) was extracted with 80% (v/v) methanol by double cold maceration. The extract was filtered through Whatman No. 1 filter paper using a vacuum pump. The collected filtrate was dried using a rotary evaporator under reduced pressure below 40°C. The resultant semisolid material was lyophilised with a final yield of 83.54 g, and stored below 20°C. The liquid–liquid partitioning was performed on the crude extract according to a standard extraction scheme (**Figure 1**). After partitioning with different solvents as shown in the scheme, *n*-hexane (1.0 g), methanol 90% (3 g), chloroform (1.69 g), ethyl acetate (1.22 g), *n*-butanol (9.63 g), and aqueous fractions (67.0 g) were obtained. The collected fractions were dried under reduced pressure at 40°C, lyophilized and stored below 20°C.

The NP (normal phase) TLC for all obtained fractions was performed using various solvent systems as mobile phase, including CH_2_Cl_2_/MeOH (70:30) with a few drops of NH_4_OH and CH_2_Cl_2_/MeOH (75:25) for the methanolic fraction; CH_2_Cl_2_/MeOH (78:22) for the chloroform and ethyl acetate fractions; *n*-hexane/CHCl_3_ (93:7) for the *n*-hexane fraction; CH_2_Cl_2_/MeOH (25:75 or 36:64) for the *n*-butanol fraction, and MeOH/CH_2_Cl_2_ (85:15) for the aqueous fraction, respectively. Developed TLC plates were examined under UV at 254 and 366 nm and after spraying with *p*-anisaldehyde

An aliquot of 0.8 g from the methanol 90% fraction was dissolved in 2 mL methanol and mixed with 1.1 g silica; the mixture was dried with nitrogen gas. The dried extract was loaded on a pre-packed Flash Grace Reveleris^®^ silica cartridge of 80 g. The compounds were eluted using a gradient from methylene chloride over ethyl acetate to methanol. Based on UV and ELSD detection, multiple subfractions were collected. All fractions were further analyzed by TLC and similar fractions were combined. In this way 14 subfractions were obtained. Flash chromatography with similar conditions was repeated when necessary. Finally, based on TLC profiling, subfractions KRM2 (200 mg), KRM3(150 mg), KRM5 (210 mg), KRM7 (200 mg), and KRM14 (110 mg) were selected for further HPLC profiling.

Similarly an aliquot of 0.8 g from the ethyl acetate fraction was subjected to flash chromatography as above. The gradient used was from methylene chloride over ethyl acetate to methanol. Based on UV and ELSD detection different subfractions (8 in total) were collected. Finally a total of 4 subfractions was obtained and subfractions KRET2 (210 mg), KRET3 (160 mg), KRET4 (100 mg), and KRET5 (270 mg) were selected for further HPLC profiling.

Likewise an aliquot of 0.8 g from the chloroform fraction was loaded on a flash column as discussed above. The compounds were eluted using a gradient from methylene chloride over ethyl acetate to methanol. Based on UV and ELSD detection different subfractions were collected. All fractions were analyzed by TLC and similar fractions were combined as described above; in this way eight subfractions were obtained. Based on TLC profiling subfractions KRCL1 (135 mg), KRCL2 (125 mg), KRCL4 (160 mg), KRCL7 (150 mg), and KRCL8 (120 mg) were selected further HPLC analysis.

Similarly an aliquot of 0.8 g from the *n*-butanol fraction was subjected to flash chromatography with a gradient from methylene chloride over ethyl acetate to methanol as previously explained. Finally, 10 subfractions were obtained. Based on TLC analysis, subfractions KRB2 (110 mg), KRB4 (127 mg), KRB5 (131 mg), KRB6 (120 mg), and KRB7 (126 mg) was selected for HPLC analysis.

The chloroform fraction and all obtained subfractions KRCL1, KRCL2, KRCL4, KRCL7, and KRCL8 were analyzed by HPLC using an optimized acetonitrile/H_2_O + 0.1% formic acid gradient, ranging from 15% acetonitrile to 80% in 50 min at a flow rate of 1 mL/min. Samples were prepared in a concentration range from 1 to 10 mg/mL in methanol. The isolation of pure compounds was performed by semi-preparative HPLC-DAD-MS using the same gradient at 3 mL/min, yielding compounds **1** (7.5 mg)**, 2** (5.2 mg), and **3** (5.2 mg).

The ethyl acetate fraction and subfractions KRET3 (250 mg), KRET4 (180 mg), and KRET5 (150 mg) were analyzed by HPLC using an optimized acetonitrile/H_2_O + 0.1% formic acid gradient ranging from 15% acetonitrile to 100% in 60 min at a flow rate of 1 mL/min. The isolation of pure compounds was performed by semi-preparative HPLC-DAD-MS using the same gradient at 3 mL/min. Compounds **3** (5.2 mg) and **4** (5.7 mg) were finally obtained.

Similarly the methanol fraction and subfractions KRM2, KRM3, KRM5, KRM7, and KRM14 were analyzed by HPLC using an optimized acetonitrile/H_2_O + 0.1% formic acid gradient ranging from 35% acetonitrile to 70% in 50 min at a flow rate of 1 mL/min. The compounds were isolated by semi-preparative HPLC-DAD-MS system using the same gradient at a flow rate of 3 mL/min. Finally compounds **2** (3.3mg), **5** (4.5 mg), and **6** (3.2 mg) were isolated.

**FIGURE 2:**
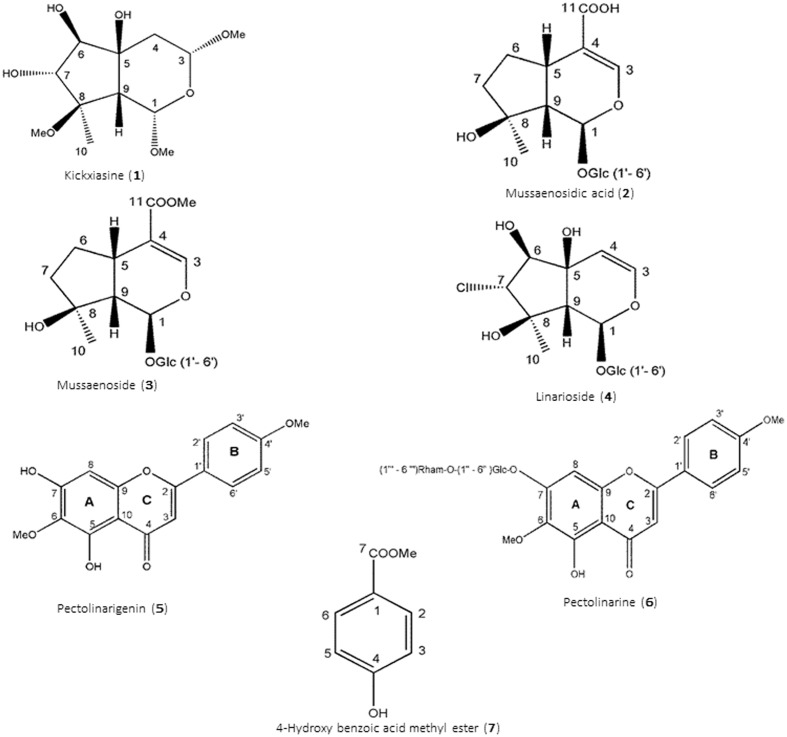
**Structures of isolated constituents (**1–7**)**.

The *n*-butanol fraction and subfraction KRB2, KRB4, KRB6, and KRB7 were analyzed by HPLC using an optimized acetonitrile/H_2_O + 0.1% formic acid gradient ranging from 39% acetonitrile to 65% in 45 min at a flow rate of 1 mL/min. Compound isolation was performed by semi-preparative HPLC-DAD-MS system using the same solvent gradient as HPLC at a flow rate of 3 mL/min. Compounds **3** (3.2 mg), **5** (3.2 mg), and **7** (3.8 mg) were finally isolated (**Figure 2**)

*Kickxiasine* (***1***). ^1^H and ^13^C NMR, Supplementary Table S1. HR-ESI-MS (positive ion mode), *m/z* 301 [M+Na]^+^ (calculated 301.1258) consistent with a molecular formula C_12_H_22_O_7_Na, or C_12_H_22_O_7_ for **1**. UV (acetonitrile/H_2_O) λ_max_ 202, 209, 216, and 225 nm. [α]^20^_D_ – 19.072 (*c* = 0.0011, MeOH).

*Mussaenosidic acid* (***2***).^1^H and ^13^C NMR, Supplementary Table S2. ESI-MS (positive ion mode), *m/z* 399 [M+Na]^+^ consistent with a molecular formula C_16_H_24_O_10_. UV (acetonitrile/H_2_O) λ_max_ 237 nm.

*Mussaenoside* (***3***). ^1^H and ^13^C NMR, Supplementary Table S3. ESI-MS (positive ion mode), *m/z* 413 [M+Na]^+^ consistent with a molecular formula C_17_H_26_O_10_. UV (acetonitrile/H_2_O) λ_max_ 237 nm.

*Linarioside* (***4***). ^1^H and ^13^C NMR, Supplementary Table S4. ESI-MS (positive ion mode), *m/z* 422 [M+Na]^+^ consistent with a molecular formula C_15_H_23_O_10_ Cl. UV (acetonitrile/H_2_O) λ_max_ 210 nm.

*Pectolinarigenin* (***5***).^1^H and ^13^C NMR, Supplementary Table S5. ESI-MS (positive ion mode), *m/z* 315 [M+H]^+^ consistent with a molecular formula C_17_H_14_O_6_. UV (acetonitrile/H_2_O) λ_max_ 275, 335 nm.

*Pectolinarin* (***6***). ^1^H and ^13^C NMR, Supplementary Table S6. ESI-MS (positive ion mode), *m/z* 623 [M+Na]^+^ consistent with a molecular formula C_29_H_34_O_15_. UV (acetonitrile/H_2_O) λ_max_ 275, 330 nm.

*4-Hydroxy benzoic acid methyl ester* (***7***). ^1^H and ^13^C NMR, Supplementary Table S7. ESI-MS (positive ion mode), *m/z* 153 [M+H]^+^ consistent with a molecular formula C_8_H_8_O_3_. UV (acetonitrile/H_2_O) λ_max_ 226, 287, 338 nm.

### Biological Evaluation

#### Antiglycation Activity

The BSA-glucose and BSA-MGO antiglycation assays were performed as reported before (Amin et al., 2016). The test protocol is attached as Supplementary Material (Amin et al., 2016).

#### Inhibition of α-Glucosidase

The α-glucosidase assay was carried out according as reported before (Amin et al., 2016). The test protocol is attached as Supplementary Material (Amin et al., 2016).

#### Inhibition of 15-Lipoxygenase Activity

The soybean LOX assay is used as an indication of anti-inflammatory activity (Muller, 1994), and therefore during the present investigation, 15-LOX from soybean was used for peroxidation of linoleic acid, and inhibition was measured as described previously with slight modifications (Malterud and Rydland, 2000). Briefly, 12.5 μL (2–0.062 mM) of sample was dissolved in DMSO, and added to 487.5 μL of enzyme solution (200 U/mL). The mixture was incubated at room temperature for 5 min. Absorbance was recorded immediately after the addition of 500 μL of substrate (250 μM linoleic acid in 0.2 M borate buffer, pH 9) every minute up to 5 min at 234 nm.

% inhibition of enzyme activity was calculated as,

%inhibition of enzyme activity=([ΔA1/Δt]−[ΔA2/Δt])/(ΔA1/Δt)×100

where Δ*A*1/Δ*t* and Δ*A*2/Δ*t* are the increase rate in absorbance at 234 nm for sample without test substance and with test substance, respectively.

#### Inhibition of Linoleic Acid Lipid Peroxidation – AAPH Assay

The inhibition of linoleic acid lipid peroxidation was studied according to Liegeois et al. (2000) and Rajic et al. (2010). Briefly, 10 μL of a 16 mM linoleic acid dispersion and 10 μL of the test compound (8-1 μM final concentration) were added to the UV cuvette containing 0.93 mL of 0.05 M phosphate buffer, pH 7.4. The oxidation reaction was initiated under air by the addition of 50 μL (40 mM) of AAPH solution. The rate of oxidation at 37°C was monitored by recording the increase of absorption at 234 nm caused by conjugated diene hydroperoxides. The results were compared to the standard inhibitor (Trolox). The AOP is defined as the slope of the curve representing the inhibition time of oxidation (T_inh_) vs. the concentration of antioxidant and is expressed in min/μM.

#### Antimicrobial Activity

Fractions and isolated compounds were evaluated for antimicrobial activity in an integrated screening panel, also including the human MRC-5 cell line to assess selectivity, as reported before (Cos et al., 2006; Mesia et al., 2008; Baldé et al., 2010; Amin et al., 2016). The IC_50_ values were determined from five 4-fold dilutions. The following positive controls were used: Tamoxifen (MRC-5, human fetal lung fibroblasts, ECACC 84100401) IC_50_ 10.48 μM; erythromycin (*Staphylococcus aureus*) IC_50_ 11.30 μM; chloramphenicol (*Escherichia coli*), IC_50_ 2.42 μM; miconazole (*Candida albicans*, B59630 Janssen strain) IC_50_ 4.70 μM; terbinafin (*Microsporum canis*, B68128 Janssen strain) IC_50_ 1.38 μM; suramine (*Trypanosoma brucei*, Squib 427) IC_50_ 0.03 μM; benznidazol (*Trypanosoma cruzi*, Tulahuen LacZ, clone C4) IC_50_ 2.13 μM; chloroquine (*Plasmodium falciparum* K1) IC_50_ 0.08 μM; miltefosine (*Leishmania infantum*, MHOM/MA/67/ITMAP263) IC_50_ 9.02 μM.

#### Statistical Analysis

IC_50_ calculations were performed using regression analysis [% inhibition vs. log (concentration)] using Sigma plot 13.0.

#### Docking Studies

AutoDock v4.2 and MGL Tools v1.5.6 (Morris et al., 2009) were used to carry out molecular docking of the isolated compounds. Crystal structures of human α-glucosidase (PDB ID 3TOP) with co-crystallized ligand acarbose and soybean LOX (PDB ID 1IK3) with co-crystallized ligand 13(S)-hydroperoxy-9(Z),11(E)-octadecadienoic acid were downloaded from the RCSB protein data bank (Bourne, 2000). The active site dimensions for each enzyme were recorded by using their co-crystallized ligands, respectively. Then, the water molecules and co-crystallized ligand were removed and hydrogen atoms and charges were added. Using ACD/ChemSketch (2015), the structures of the isolated compounds were drawn and 3D optimized. Molecular docking was performed using Lamarckian Genetic Algorithm embedded in AutoDock v4.2. A total number of 100 different poses were generated and clustered according to their RMSD values. Each cluster was carefully visualized in Discovery Studio Visualizer (Berman et al., 2005) and putative binding modes were selected accordingly.

## Results and Discussion

### Structure Elucidation

Structures of the isolated compounds from *Kickxia ramosissima* were elucidated using ^1^H- and ^13^C-NMR (including DEPT-135 and DEPT-90) and 2D-NMR (COSY, HSQC, and HMBC) spectroscopy (all assignments and spectra are attached as Supplementary Material; Tables S1–S7 and Figures S1–S18, respectively). The molecular ion was derived from the mass spectra obtained with the semi-preparative HPLC-DAD-MS system, and the UV absorption maxima from the diode array detection.

The ^1^H-spectrum of compound **1** showed signals due to one methyl, one methylene, six methines, and three methoxyls. The methylene signals at 1.51 ppm (dd, *J* = 14.0, 8.4) and 2.13 ppm (m) were assigned to H-4. A methine at 4.88 ppm (dd, *J* = 5.3, 8.4 Hz) was attributed to H-3. A methine signal at 2.05 ppm (d, *J* = 7.3 Hz, H-9) was coupled with another methine at 4.94 ppm (d, *J* = 7.3 Hz, H-1), an upfield signal that was indicative of substitution with oxygen. Finally two methine protons showed coupled doublets at 3.77 and 3.93 ppm (*J* = 4.3 Hz), assigned to H-7 and H-6, respectively. The methyl group at 1.35 ppm (s, H-10) was attached to an oxygen-bearing quaternary carbon as determined by HMBC correlations. The ^13^C-NMR spectrum showed 12 carbon signals. The signals at 98.7 and 98.8 ppm were assigned to carbons C-1 and C-3, respectively. In the cyclopentane ring the signals at 75.9 and 77.3 ppm were attributed to C-6 and C-7, respectively. In the same ring the signals at 83.2, 77.4, and 60.3 ppm were assigned to C-8, C-5, and C-9, respectively. The methyl group (C-10) showed a resonance signal at 17.1 ppm. Three methoxyl groups in positions 1, 3, and 8 showed signals at 50.3, 55.6, and 56.3 ppm, respectively. The protons of these methoxyl groups were correlated in the HMBC spectrum with C-1, C-3 and C-8. The methyl group (C-10) at 1.35 ppm was attached to C-8, which was also established by HMBC correlations of the methyl protons with C-8 and C-9. HMBC (Supplementary Figure S6) correlations from C-1 to the methoxyl group attached to C-8 and to H-9, and from C-6 to H-10 confirmed that the cyclopentane ring was substituted by a methyl group (C-10) and a methoxyl group. Similarly, in the second ring, the signals at 99.7, 99.8, and 40.9 ppm were assigned to C-1, C-3, and C-4, respectively. Also the HMBC correlations from H-1 to the methoxyl group attached to C-3 and from C-8 to the methoxyl group attached to C-1 confirmed the position of these methoxyl groups at C-1 and C-3.

The relative configuration was assigned on the basis of chemical shifts and coupling constants reported for related compounds (Morota et al., 1990; Awale et al., 2005; Ahmad et al., 2006), and by analogy with linarioside (compound **4**). The ^13^C-NMR chemical shifts and 2D NMR experiments support the structure of compound **1** as a new compound for which the name kickxiasine was adopted. Kickxiasine is reported here for the first time from nature. Although a number of polymethoxylated iridoids have been reported from different medicinal plants, for instance *Tabebuia avellanedae* (Awale et al., 2005), *Rehmannia glutinosa* (Morota et al., 1990; Liu et al., 2014), and *Gonocaryum calleryanum* (Kaneko et al., 1995), however, the possibility that compound **1** is an artifact formed during extraction and isolation cannot be excluded.

Compound **2** showed typical signals of an iridoid glucoside. Further comprehensive analysis showed that compound **2** was mussaenosidic acid, which has been reported from various medicinal plants including *Pedicularis kerneri* (Venditti et al., 2016), *Kickxia elatine* (L.) Dum., *Kickxia spuria* (L.) Dum. (Handjieva et al., 1995), *Lagochilus ilicifolius* (Guangzhou et al., 2012), and *Vitex negundo* (Sehgal et al., 1982). However, mussaenosidic acid is reported here for the first time in *Kickxia ramosissima*.

Compound **3** was identified mussaenoside, which is the methyl ester of mussaenosidic acid (**2**). Although this compound has been reported before, still the possibility that it is an artifact formed during extraction procedures in which methanol is used cannot be excluded. Mussaenoside is a common iridoid and has been isolated from a number of plants species, for instance *Melampyrum* sp. (Damtoft et al., 1984), *Bellardia trixago* (Ersoz et al., 1998), and *Mussaenda incana* (Dinda et al., 2005). This is the first report of its occurrence in *K. ramosissima*.

Compound **4** was identified as linarioside, which was the first example of a chlorine containing iridoid glucoside in nature isolated from *Linaria japonica* (Kitagawa et al., 1973; Otsuka, 1993). A detailed investigation proved that linarioside is a naturally occurring compound and was not formed as an artifact during the extraction process. Afterward a number of other medicinal plants were reported to contain linarioside. These include *Cymbalaria muralis* (Kapoor and Reisch, 1974), *Asystasia bella* (Demuth et al., 1989), *Linaria aegyptiaca* (Ferhat et al., 2010), and *Linaria genistifolia* (Ilieva et al., 1992). Here linarioside is reported for the first time in *K. ramosissima.*

Compound **5** was identified as pectolinarigenin which is a common flavonoid reported from various medicinal plants including *Linaria reflexa* (Cheriet et al., 2014), *Cirsium chanroenicum* (Lim et al., 2008), and also from *K. ramosissima* (Singh and Prakash, 1987).

Compound **6** was identified as pectolinarine, which is glycosylated flavonoid isolated before from a number of plants species including *Cirsium japonicum* (Liu et al., 2007), *Cirsium chanroenicum* (Lim et al., 2008), *Kickxia abhaica* (Al-Rehaily et al., 2006), and *K. ramosissima (*Ahmad et al., 2006).

Finally compound **7** was identified as 4-hydroxy-benzoic acid methyl ester, occurring in many medicinal plant species, for instance *Vitex rotundifolia* (Yoshioka et al., 2004) and *Houttuynia cordata* (Jong and Jean, 1993). This is, however, the first report of its presence in *K. ramosissima.*

### Biological Activities

#### Antiglycation Activity

As *K. ramosissima* is well known for its use in diabetic conditions, crude extracts and isolated compounds were tested for antiglycation activity. Glucose-mediated protein glycation models are generally used to determine inhibition of the formation of AGEs. However, AGE-protein adducts can be formed both under oxidative and non-oxidative conditions, and therefore AGEs inhibitors should be differentiated from common antioxidants (Rahbar, 2007). Evaluation of the inhibition of AGEs formation starting from glucose and BSA involves all possible mechanisms. However, RCS such as glyoxal and methyl glyoxal that are intermediate products of the Maillard reaction, have already undergone the oxidation processes starting from glucose. Inhibition of their reaction with BSA is representative of non-oxidative glycation reactions.

The ethyl acetate fraction was highly active in the BSA-glucose assay (IC_50_ 88 μg/mL), whereas a moderate antiglycation activity was observed for the *n*-butanol (36% inhibition) and methanol (32% inhibition) fractions at 100 μg/mL. A mild activity (20% inhibition) was seen for the chloroform fraction (**Table 1**). In the case of iridoids (compounds **1**–**4**) only mild inhibition of protein glycation was noticed (**Table 2**). In particular compound **2** was most active (35% inhibition at the highest test concentration of 3 mM, compared to an IC_50_ of 1.75 mM of the positive control substance aminoguanidine), followed by compound **3** (28% inhibition) and compound **1** (26% inhibition). Compound **4** did not present any activity. A nearly similar moderate trend was seen in the BSA-MGO assay. There has only been one previous report on weak inhibition of protein glycation by iridoids (West et al., 2014). Contrary to iridoids, the isolated flavonoids **5** (IC_50_ 0.79 mM) and **6** (IC_50_ 2.29 mM) were more active, which was consistent with the high activity of the ethyl acetate fraction. The fact that the glycosylated compound **6** was less active than the aglycone **5** is in agreement with glycosylation effects of flavonoids previously reported (Matsuda et al., 2003). This inhibition was mainly due to the non-oxidative mode of inhibition as obvious in the BSA-MGO assay, since the IC_50_ values for compounds **5** (IC_50_ 0.19 mM) and **6** (IC_50_ 0.13 mM) were lower than in the glucose-BSA assay. Finally the benzoic acid derivative **7** did not present any inhibition in both models. The anti-glycation activity of most of the isolated constituents was rather moderate compared to, e.g., the ethyl acetate fraction, which could be explained by synergistic effects.

**Table 1:** Anti-glycation activity in the BSA-glucose assay of *Kickxia ramosissima* fractions.

Sample	% inhibition^a^	IC_50_ (μg/mL)
Total Extract	5	–
MeOH 90%	32	–
Chloroform	20	–
Ethyl acetate	64	88
*n*-Butanol	36	–
Aminoguanidine		199


*^a^At 100 μg/mL.*

**Table 2:** Antiglycation activity in the BSA-glucose and BSA-MGO assays of isolated constituents **1–7**.

Compound	Protein glycation			
	
	BSA-Glucose		BSA-MGO	
		
	% inhibition^a^	IC_50_ (mM)	% inhibition^b^	IC_50_ (mM)
1	26		31	
2	35		23	
3	28		24	
4	no activity		9	
5		0.79		0.19
6		2.29		0.13
7	no activity		no activity	
Aminoguanidine		1.75		0.15
Quercetin		0.23		–


*BSA, bovine serum albumin; MGO, methylglyoxal.*

*^a^At 3 mM final concentration.*

*^b^At 1.3 mM final concentration.*

#### Inhibition of α-Glucosidase

In a sequel of testing the isolated compounds for their antidiabetic potential, the assay on inhibition of α-glucosidase was performed. It was noticed that iridoids presented only mild levels of inhibition (**Table 3**). The highest inhibition (29% inhibition at the highest test concentration of 2 mM, compared to an IC_50_ of 0.26 mM for the positive control substance acarbose) was seen for compound **4,** followed by compound **2** (15% inhibition) and compound **3** (9% inhibition). The newly isolated compound **1** did not present any activity. In general a number of reports suggested the potential of iridoids as α-glucosidase inhibitors, but mainly moderate inhibition was noticed (Hua et al., 2014; Lin et al., 2015). The flavonoids, however, presented better activity compared to tested iridoids. Compound **5** showed the highest activity (IC_50_ 0.23 mM, which is similar to acarbose), whereas its glycosylated counterpart compound **6** presented moderate α-glucosidase inhibition (48% inhibition at the highest test concentration). The flavonoids are generally known for their multiple therapeutic effects including α-glucosidase inhibitory activity (Tadera et al., 2006; Hong et al., 2013). A moderate α-glucosidase inhibition (47% inhibition Vs Acarbose 0.26 mM) was observed for the benzoic acid derivative **7**. Based on our findings, we could conclude that mainly compound **5** may to some extent contribute to the traditional use of *K. ramosissima* in diabetic conditions.

**Table 3:** α-glucosidase inhibitory activity of isolated constituents **1–7**.

Compound	% inhibition^a^	IC_50_ (mM)
1	no activity	-
2	15	-
3	9	-
4	29	-
5	-	0.23
6	48	-
7	47	-
Acarbose		0.26


*^a^At 2 mM final concentration.*

#### Inhibition of 15-Lipoxygenase Activity

Iridoids are particularly known for their anti-inflammatory activities (Park et al., 2010; Gousiadou et al., 2013). As the major constituents of *Kickxia* were iridoids, it was therefore considered interesting to test the isolated iridoids for inhibition of their 15-LOX potential. Since also flavonoids are reported to possess LOX inhibitory potential (Lyckander and Malterud, 1996; Malterud and Rydland, 2000), it was therefore decided to investigate the isolated flavonoids in the 15-LOX assay as well. The highest inhibition among the iridoids was observed for compound **2** (IC_50_ 0.22 mM, compared to 0.14 mM for the positive control substance quercetin) (**Table 4**). Likewise in the case of flavonoids compound **6** (IC_50_ 0.25 mM) presented the highest inhibition. The benzoic acid derivative **7** presented only a moderate level of inhibition (30% inhibition at the highest test concentration). Based on these findings we could to some extend confirm the possible role of iridoids and flavonoids as anti-inflammatory agents in *K. ramosissima*.

**Table 4:** 15-LOX inhibitory activity and inhibition of linoleic acid peroxidation (AAPH assay) by isolated constituents **1–7**.

Compound	15-LOX assay	AAPH assay	
	
	% inhibition^a^	IC_50_ (mM)	AOP (min/μM)^b^
1	30	–	0.3
2	–	0.22	0.2
3	37	–	0.1
4	8	–	Not tested
5	46	–	0.2
6	–	0.25	0.3
7	30	–	Not active
Quercetin	–	0.14	–
Trolox	–	–	14.12^c^


*AOP, antioxidant power.*

*^a^At 0.312 mM final concentration.*

*^b^At 100 μM.*

*^c^At 8 μM.*

#### Inhibition of Linoleic Acid Lipid Peroxidation – AAPH Assay

**FIGURE 3:**
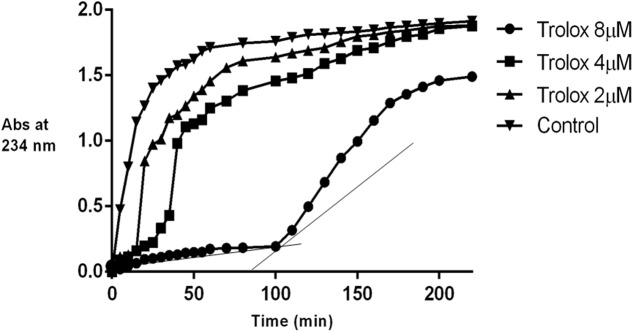
**Effect of Trolox on AAPH induced linoleic acid oxidation**.

**FIGURE 4:**
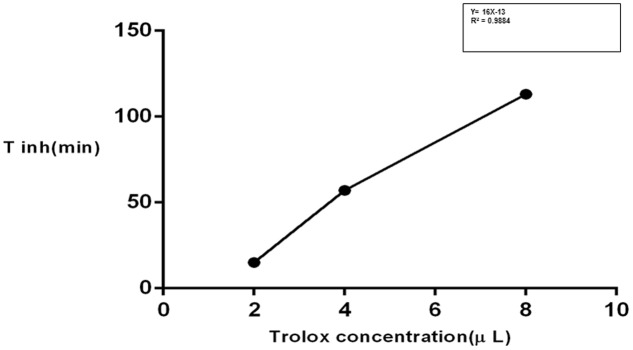
**Time of inhibition (T_inh_) as a measure of time**.

The isolated iridoids (**1–4**) were evaluated for inhibition of AAPH induced linoleic acid peroxidation. While compound **4** was not tested due to limited amount, compounds 1**–3** displayed week activity in the range of 0.1–0.3 min/μM, compared to standard Trolox (14.12 min/μM) (**Table 4** and **Figures 3**, **4**). Our findings were in agreement with week activities reported previously for mussaenoside and mussaenosidic acid (Gousiadou et al., 2013). Isolated flavonoids (**5–6**) only weakly active in the range of 0.2–0.3 min/μM, compared to standard Trolox (14.12 min/μM) (**Table 4** and **Figures 3**, **4**). A similar trend had been reported for different types of flavonoids (Peyrat-Maillard et al., 2003). Compound **7** did not present any activity.

### Antimicrobial Activity

The crude extracts were evaluated for antibacterial and antifungal activities. The highest levels of antibacterial and antifungal activities were observed for n the *n*-hexane fraction, i.e., an IC_50_ of 8.0 μg/mL against *S. aureus* and 24.4 μg/mL against *M. canis*. None of the other fractions were found active in the test range. Compounds **2**–**7** were evaluated in an integrated screening panel for antimicrobial activity. Compound **1** was not tested due to the limited amount available. None of the isolated iridoids presented antimicrobial activity in the test range. Although few iridoids have been reported to exhibit antimicrobial activities (Ishiguro et al., 1983; Davini et al., 1986), such activity is surely related to certain structural features. A number of investigations have reported antimicrobial activity of iridoid-rich fractions of medicinal plants, for instance *Morinda citrifolia* (noni) fruits (West et al., 2012). Nevertheless, iridoids can be considered as prodrugs that may be deglycosylated and activated after oral administration. Flavonoids are well known for their antimicrobial activities (Mohanty et al., 2015; Xie et al., 2015). Also during the current investigation, pectolinarigenin (**5**) was found active against *Staphylococcus aureus* (IC_50_ 49.8 μM). This compound also showed weak antiprotozoal activities against *Plasmodium falciparum* K1 (IC_50_ 41.8 μM) and *Trypanosoma cruzi* (IC_50_ 32.0 μM). Moreover it was not cytotoxic against MRC-5 cells.

### Docking Studies

**FIGURE 5:**
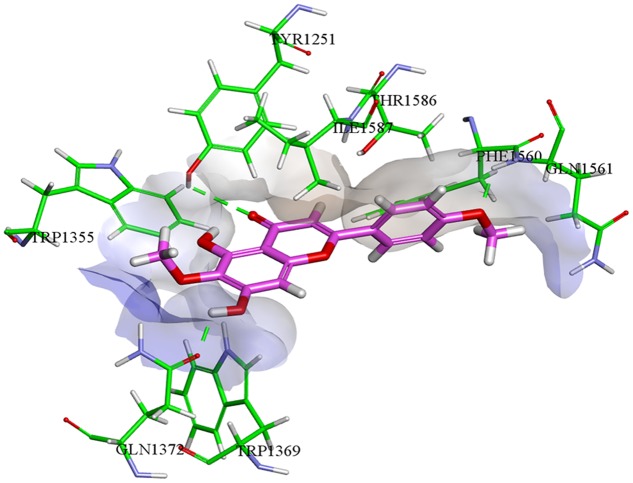
**Compound **5** (colored magenta) inside α-glucosidase active binding site (colored green)**.

Molecular docking studies for isolated compounds were performed for inhibition of α-glucosidase and soybean LOX (15-Lox) (**Table 5**). Inside the α-glucosidase active site different binding affinities were determined for the isolated compounds. No good correlation was determined for the binding affinities and *in vitro* results. However, comparatively less binding affinity was determined for compounds **1** and **7** as compared to other isolated compounds. Lower binding affinities of compounds **1** and **7** are synchronized with the *in vitro* results. Compound **5** being highly potent against α-glucosidase *in vitro*, was found computationally to give hydrogen bonding interactions with residues Tyr1251, Gln1372, and Gln1561. The binding pose determined for compound **5** was different from the binding pose of the standard acarbose. For compound **5**, a binding affinity of -7.01 kcal mol^-1^ was determined in case of α-glucosidase. The putative binding mode of compound **5** inside α-glucosidase is given in **Figure 5**.

**Table 5:** Binding free energy of the isolated compounds as predicted by docking studies.

Compound no.	Binding free energies (kcal mol^-1^)	
	
	α-glucosidase	Lipoxygenase
1	-5.47	-6.23
2	-9.43	-9.21
3	-8.46	-8.03
4	-8.64	-8.20
5	-7.01	-7.56
6	-10.21	-1.12
7	-4.65	-5.19


**FIGURE 6:**
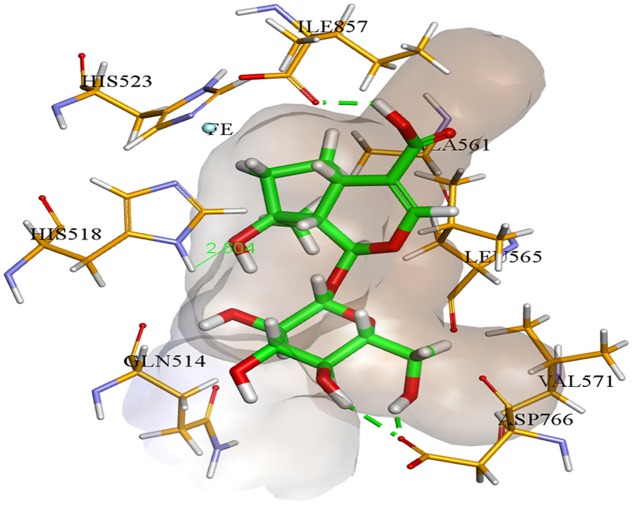
**Compound **2** (colored green) inside soybean lipoxygenase active binding site (colored brown)**.

In case of soybean LOX, compound **2** was found to be most potent inhibitor. It was found to bind inside the active site of soybean LOX with a binding affinity of -9.21 kcal mol^-1^. Compound **2** gave hydrogen bonding interactions with residues His518, Asp766, and Ile857. Similarly, hydrogen bonding interaction with residue Ile857 was common to hydrogen bonding interaction of the co-crystallized ligand 13(S)-hydroperoxy-9(Z), 11(E)-octadecadienoic acid. The putative binding mode of compound **2** inside soybean LOX is given in **Figure 6**. Based on our findings we could demonstrate the moderate antidiabetic, anti-inflammatory, and antimicrobial activity of some constituents of *K. ramosissima* and therefore at least in part confirm its antidiabetic use in traditional medicine.

## Author Contributions

AA has carried out the isolation, structure elucidation and biological evaluation of all extracts and compounds. ET has assisted with the structure elucidation (NMR) and the antiglycation assays. KF has assisted with the isolation and structure elucidation (MS). JI was responsible for the *in silico* evaluation and docking studies. PC and LM have supervised the antimicrobial evaluation in their laboratory. VE has assisted with the structure elucidation (NMR). SA and LP have supervised the Ph.D. project of AA.

## Conflict of Interest Statement

The authors declare that the research was conducted in the absence of any commercial or financial relationships that could be construed as a potential conflict of interest.

## Abbreviations

AAPH: 2,2′-azobis-(2-methyl-propionamidine)-dihydrochloride
AGEs: advanced glycation endproducts
AOP: antioxidant power
BSA: bovine serum albumin
LOX: lipoxygenase
MGO: methyl glyoxal
PMM: peripheral murine macrophages
RCS: reactive carbonyl substances
