# Supplementary material for: In Vitro and In Silico Antidiabetic and Antimicrobial Evaluation of Constituents from Kickxia ramosissima (Nanorrhinum ramosissimum)
Source: Front Pharmacol. 2017 May 1;8:232. doi: 10.3389/fphar.2017.00232 (PMC5410595; doi:10.3389/fphar.2017.00232)
Supplement: Supplementary file 1 [file Data_Sheet_1.PDF]

In vitro and *in silico* antidiabetic and antimicrobial evaluation of constituents from *Kickxia ramosissima* (*Nanorrhinum ramosissimum*)

Adnan Amin [1], Emmy Tuenter [1], Kenn Foubert [1], Jamhsed Iqbal [3], Paul Cos [2], Louis Maes [2], Vassiliki Exarchou [1], Sandra Apers [1] and Luc Pieters [1*]

[a] Natural Products & Food Research and Analysis (NatuRA), Department of Pharmaceutical Sciences, University of Antwerp, Universiteitsplein 1, B-2610 Antwerp, Belgium.

[b] Laboratory of Microbiology, Parasitology and Hygiene (LMPH), Faculty of Pharmaceutical, Biomedical and Veterinary Sciences, University of Antwerp, Universiteitsplein 1, B-2610 Antwerp, Belgium.

[c] Centre for Advanced Drug Research, COMSATS Institute of Information Technology, Abbottabad, Pakistan.

*** Corresponding author**
Prof. Dr. Luc Pieters
Natural Products & Food Research and Analysis (NatuRA)
Department of Pharmaceutical Sciences
University of Antwerp
Universiteitsplein 1
2610 Antwerp – Belgium
luc.pieters@uantwerpen.be
+32-3 265 27 15

Antiglycation assay (BSA-Glucose assay)

In the BSA-glucose assay the prevention of AGEs formation is investigated by determining the fluorescence of the complex formed after incubation of a sugar (glucose) with protein (BSA) in the presence or absence of test compounds. The antiglycation assay was performed according to the method developed by Matsuura *et al.* (2002) with minor modifications. The reaction mixture (300 µL) contained bovine serum albumin (BSA) (10 mg/ml, 135 µL), D-glucose (500 mM, 135 µL) dissolved in phosphate buffer (50 mM, pH 7.4, containing sodium azide (0.02%, added to prevent bacterial growth) and test compounds (30 µL), at different final concentrations (1.5 – 0.023 mM for isolated constituents or 100–1.17 µg/ml for extracts) dissolved in 100 % DMSO. The mixtures were incubated at 60°C for 48 h. After incubation, the reaction mixture was allowed to cool down at room temperature. Then 100 µL reaction mixture was transferred to a new plastic tube (1.5 mL) and the reaction was stopped by adding 10 µL of 100% (w/v) trichloroacetic acid (TCA) and to precipitate proteins. The TCA-added mixture was kept at 4°C for 10 min, followed by centrifugation (14000 rpm, 4 °C, 4 min). The supernatant containing unreacted D-glucose, test sample and interfering substances was discarded, whereas the precipitate containing AGEs-BSA was redissolved with 0.8 mL alkaline PBS (137 mM NaCl, 8.1 mM Na_2HPO_4, 2.68 mM KCl, 1.47 mM KH_2PO_4, pH 10). The change in fluorescence intensity (excitation 335 nm, emission 385 nm; excitation 370, emission 440) due to formation of AGEs was monitored by spectrofluorometry (Tecan™ Infinite M200, Giessen, The Netherlands). In order to eliminate interference by autofluorescence of the test compounds, a parallel incubation of test substance with BSA at 60°C without D-glucose was performed for all samples. Quercetin (0.5- 0.0312 mM final concentration) and aminoguanidine (3 - 0.046 mM final concentration) were used as reference compounds.

The AGEs inhibition was calculated as

$$\% \text{ inhibition} = \{1- (F_{BSA + glucose + test\ substance} – F_{BSA\ +\ test\ substance}) / (F_{BSA + glucose} – F_{BSA})\} \times 100$$

where F is the fluorescence intensity. The concentration required for 50% inhibition (IC_{50}) was calculated using Sigma plot® 13.0.

Protein-glyoxal interaction (BSA-MGO assay)

The antiglycation assay using methylgloxyl (MGO) was performed according to the method developed by Peng *et al*. (2007) with slight modifications. Briefly, methylglyoxal (135 µL, 5.75 mM) was incubated with BSA (135 µL, 10 mg/mL) dissolved in phosphate buffer (50 mM, pH 7.4, containing sodium azide (0.02%), added to prevent bacterial growth) and test compounds (30 µL) at different final concentrations (1.5 – 0.023 mM) in 100% DMSO. The reaction mixture was incubated at 37 °C for one week. Control solutions only contained methylglyoxal (135 µL, 5.75 mM) BSA (135 µL, 1 0mg/mL) and DMSO (100%) only. The blank samples of similar composition were also prepared simultaneously and kept at 4 °C for one week. The change in fluorescence intensity (excitation 335 nm, emission 385 nm; excitation 370, emission 440) due to formation of AGEs was monitored by spectrofluorometry (Tecan® Infinite M200, Giessen, The Netherlands). Aminoguanidine (3-0.046 mM final concentration) was used as reference compound. The AGEs inhibition was calculated as

% AGEs inhibition = [1–(S–Sb)/(C–Cb)] x 100

where S and C were fluorescence of test samples (in DMSO) and control (test mixtures containing only DMSO) incubated at 37 °C, and where Sb and Cb were fluorescence for samples incubated at 4 °C. The concentration required for 50 % inhibition (IC_{50}) was calculated using Sigma plot® 13.0.

Inhibition of α-glucosidase.

α-Glucosidase inhibitory activity was assayed according to method adopted by Choudhary *et al*. (2010) with some modifications. Briefly, 50 µL of a solution of α-glucosidase from *Saccharomyces cerevisiae* (0.2 U/mL in 0.1 M phosphate buffer at pH 6.8, Sigma-Aldrich, St. Louis, MO, USA) was incubated with 20 µL of test compounds or extracts at different concentrations (ranging from 6 mM – 0.372 mM in 25% DMSO for isolated constituents or from 5 mg to 0.039 mg/mL for extracts) at 37 °C for 10 min. Then 50 µl of the substrate, *p*-nitrophenyl-α-D-glucopyranoside (0.7 mM stock, final concentration 0.29 mM) was added to the reaction mixture and incubated again for 30 min at 37 °C. The final concentrations of test substances in the reaction mixture ranged from 1 – 0.062 mM for isolated constituents, and from 834 µg to 6.5 µg/mL for extracts. The reaction was stopped by adding Na_2CO_3 (100 µl, 200 mM stock) solution and the absorbance was measured at 400 nm. Final DMSO concentration remained below 7.5% during the assay. Acarbose (1 – 0.031 mM final concentration) was used as reference compound.

The % inhibition was calculated using the following formula:

$$\% \text{ Inhibition} = 100 - [OD_{(\text{test sample})} / OD_{(\text{control})}] \times 100$$

Alpha-glucosidase inhibition was categorized as mild (1-25%), moderate (26-50%), high (50-70%), and very high (70-100%).

References

Matsuura, N., Aradate, T., Sasaki, C., Kojima, H., Ohara, M. & Hasegawa, J., 2002. Screening system for the Maillard reaction inhibitor from natural product extracts. Journal of Health Sciences and Surveillance system, 48, *520-526*.

Peng, X., Zheng, Z., Cheng, K. W., Shan, F, Ren, G. X., Chen, F. & Wang, M., 2007. Inhibitory effect of mungbean extract and its constituents vitexin and iso-vitexin on the formation of advanced glycation end products. Food Chemistry 106, 475–481.

Choudhary MI, Shah SA, Atta-ur-Rahman, Khan SN, Khan MT, 2010. α- glucosidase and tyrosinase inhibitors from fungal hydroxylation of tibolone and hydroxytibolones. *Steroids* **75**: 956–966.

Table S1. ^{1}H- and ^{13}C-NMR assignments of Kickxiasine (**1**) recorded in MeOH-d_4

Table S2. ^{1}H- and ^{13}C-NMR assignments of Mussaenosidic acid (**2**) recorded in MeOH-d_4

Table S3. ^{1}H- and ^{13}C-NMR assignments of Mussaenoside (**3**) recorded in MeOH-d_4

Table S4. ^{1}H- and ^{13}C-NMR assignments of Linarioside (**4**) recorded in MeOH-d_4

Table S5. ^{1}H- and ^{13}C-NMR assignments of Pectolinarigenin (**5**) recorded in MeOH-d_4

Table S6. ^{1}H- and ^{13}C-NMR assignments of Pectolinarin (**6**) recorded in MeOH-d_4

Table S7. ^{1}H- and ^{13}C-NMR assignments of 4-Hydroxy benzoic acid methyl ester (**7**) recorded in MeOH-d_4

Fig. S1. ^{13}C-NMR, DEPT-135 and DEPT-90 spectra of Kickxiasine (**1**) recorded in MeOH-d$_4$

Fig. S2. ^{1}H-NMR spectrum of Kickxiasine (**1**) recorded in MeOH-d$_4$

Fig. S3. ^{1}H-NMR spectrum of Kickxiasine (**1**) recorded in MeOH-d$_4$

Fig. S4. COSY spectrum of Kickxiasine (**1**) recorded in MeOH-d$_4$

Fig. S5. HSQC spectrum of Kickxiasine (**1**) recorded in MeOH-d$_4$

Fig. S6. HMBC spectrum of Kickxiasine (**1**) recorded in MeOH-d$_4$

Fig. S7. ^{13}C-NMR, DEPT-135 and DEPT-90 spectra of Mussaenosidic acid (**2**) recorded D_2O

Fig. S8. ^{1}H-NMR spectrum of Mussaenosidic acid (**2**) recorded in D_2O

Fig. S9. ^{13}C-NMR, DEPT-135 and DEPT-90 spectra of Mussaenoside (**3**) recorded in MeOH-d$_4$

Fig. S10. ^{1}H-NMR spectrum of Mussaenoside (**3**) recorded in MeOH-d$_4$

Fig. S11. ^{13}C-NMR, DEPT-135 and DEPT-90 spectra of Linarioside recorded in D_2O

Fig. S12. ^{1}H-NMR spectrum of Linarioside (**4**) recorded in D_2O

Fig. S13. ^{13}C-NMR, DEPT-135 and DEPT-90 spectra of Pectolinarigenin (**5**) recorded in $CDCl_3$

Fig. S14. ^{1}H-NMR spectrum of Pectolinarigenin (**5**) recorded in $CDCl_3$

Fig. S15. ^{13}C-NMR, DEPT-135 and DEPT-90 spectra of Pectolinarin(**6**) recorded in DMSO-d_6

Fig. S16. ^{1}H-NMR spectrum of Pectolinarin (**6**) recorded in DMSO-d_6

Fig. S17. ^{13}C-NMR, DEPT-135 and DEPT-90 spectra of 4-Hydroxy benzoic acid methyl ester (**7**) recorded in MeOH-d$_4$

Fig. S18. ^{1}H-NMR spectrum of 4-Hydroxy benzoic acid methyl ester (**7**) recorded in MeOH-d$_4$.

Table S1. ^{1}H and ^{13}C NMR assignments for compound (**1**) recorded in MeOH-d_4

**Position**	**δ_H (ppm); multiplicity; *J* (Hz)**	**δ_C (ppm)**
**1**	4.94; d; *J*=7.3	99.7
**3**	4.88; dd; *J*=8.4, 5.3	99.8
**4_a**	2.13; m	40.9
**4_b**	1.51; dd; *J*= 14.0, 8.4*	
**5**		77.4
**6**	3.93 ; d; *J*=4.3	75.9
**7**	3.76; d; *J*=4.3	77.3
**8**		83.2
**9**	2.05; d, *J*= 7.3	60.3
**10**	1.35; s	17.1
**OMe (C-1)**	3.47; d; *J*=6.7	50.3
**OMe (C-3)**	3.47; s	55.6
**OMe (C-8)**	3.36; s	56.3

* Multiplicity and coupling constants were calculated on a spectrum recorded in acetone-d_6 because of overlapping with the HDO peak in the spectrum recorded in MeOH-d_4.

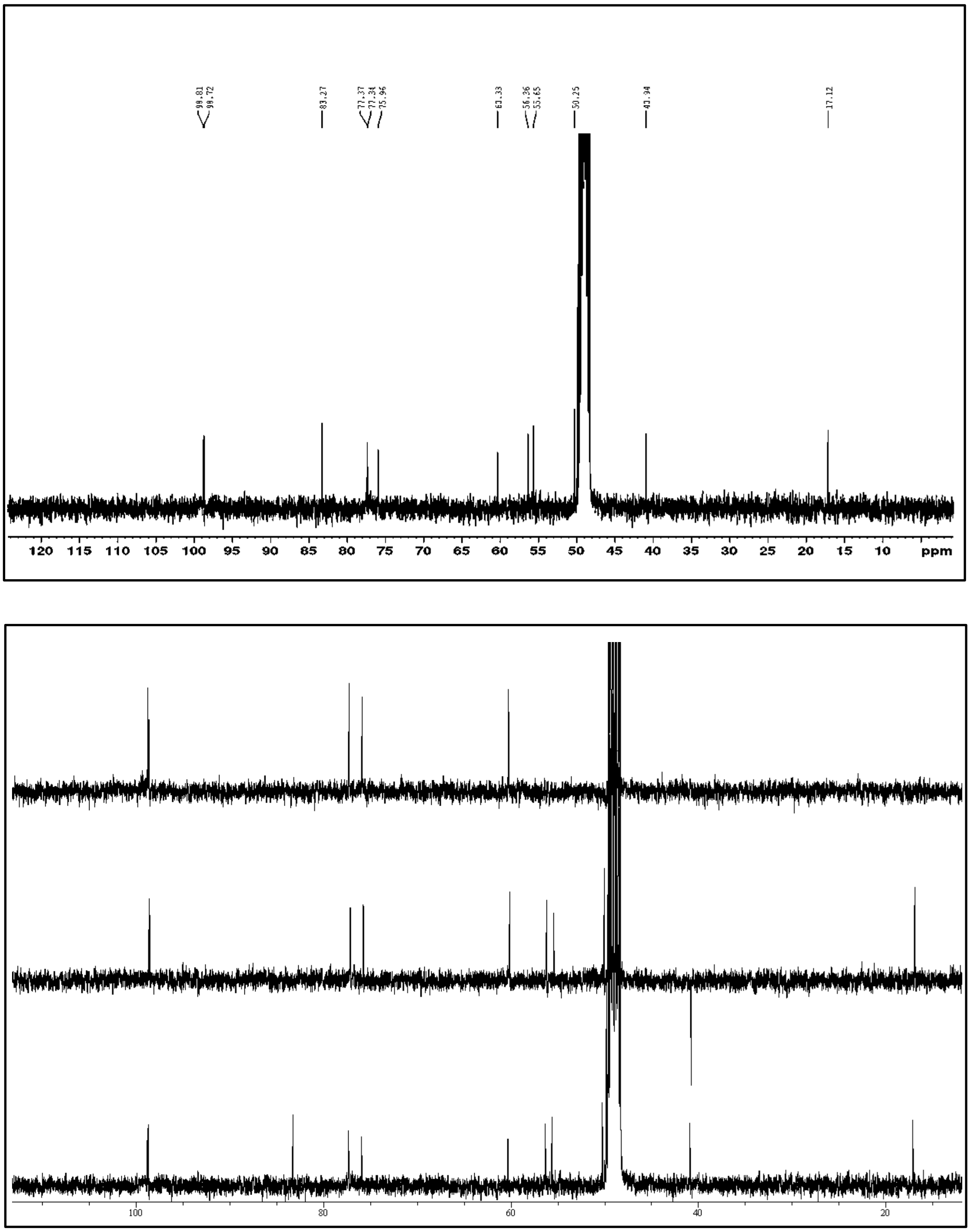

Figure S1. ^{13}C-NMR, DEPT-135 and DEPT-90 spectra of compound **1** in methanol-d_4.

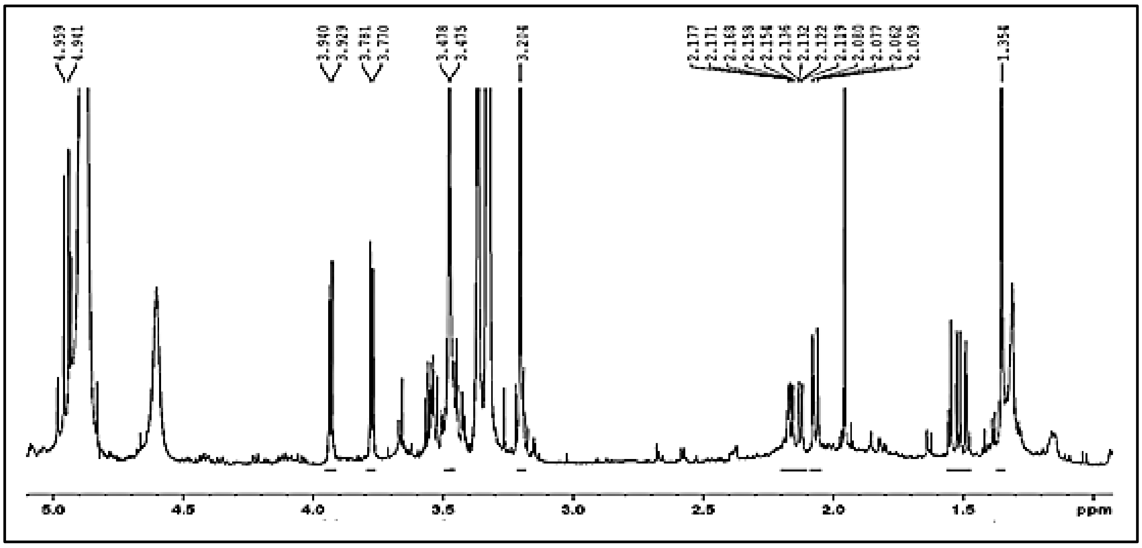

Figure S2. ^{1}H-NMR spectrum of compound **1** in methanol-d_4

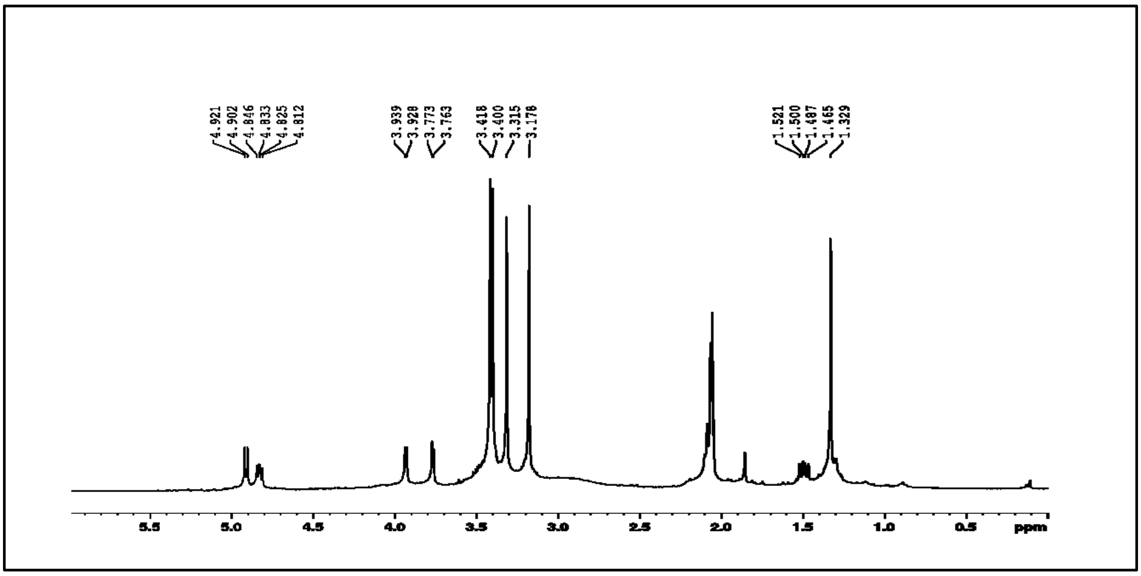

Figure S3. ^{1}H-NMR spectrum of compound **1** in acetone-d_6

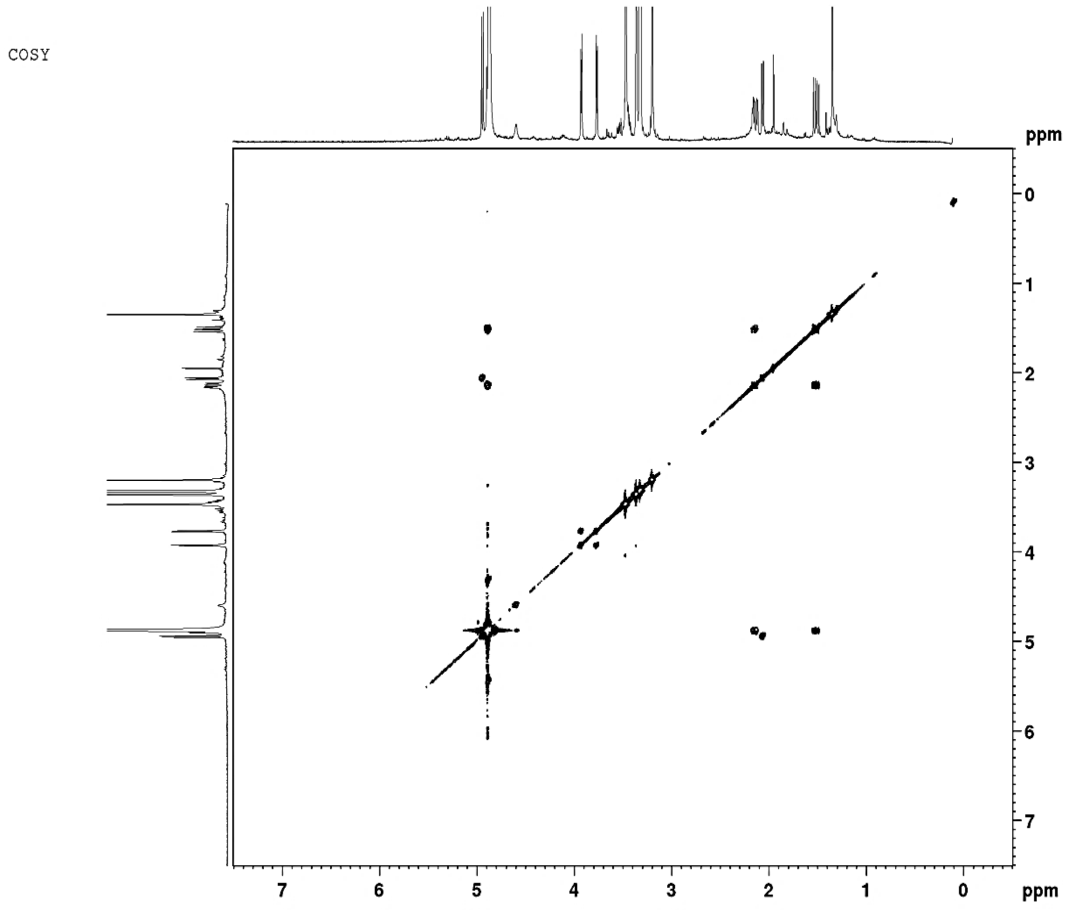

Figure S4. COSY spectrum of compound **1**

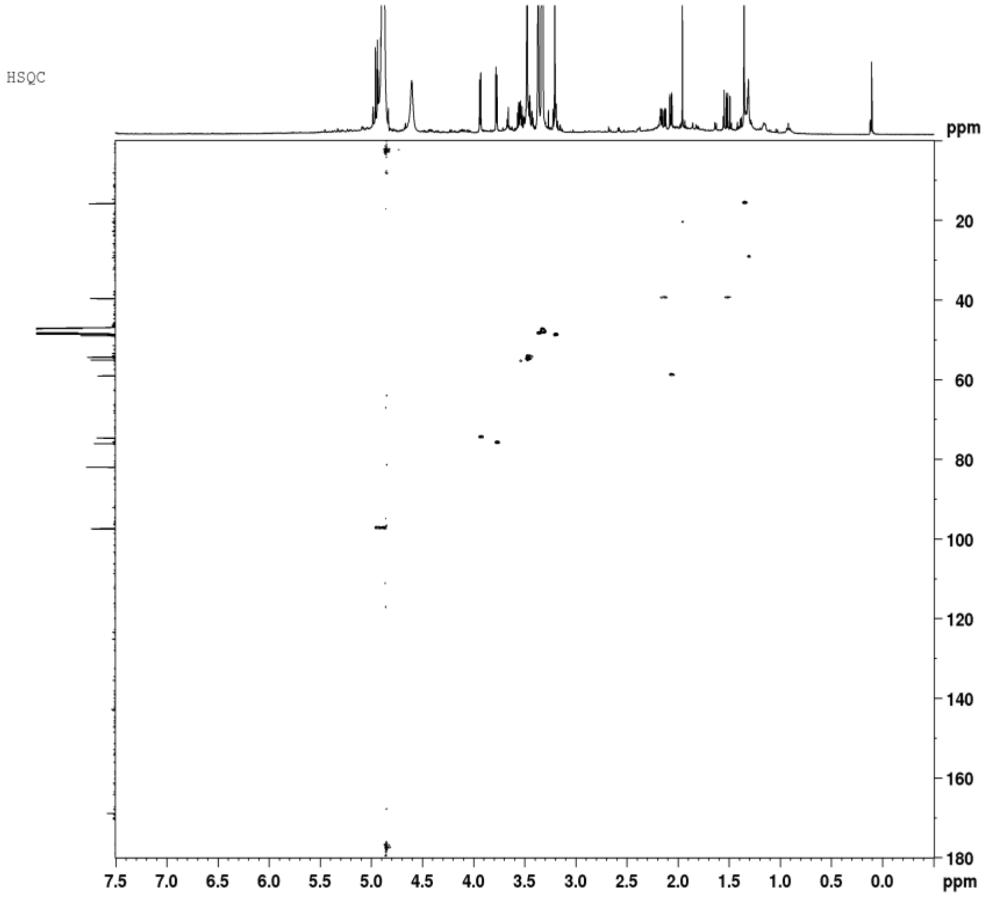

Figure S5. HSQC spectrum of compound **1**

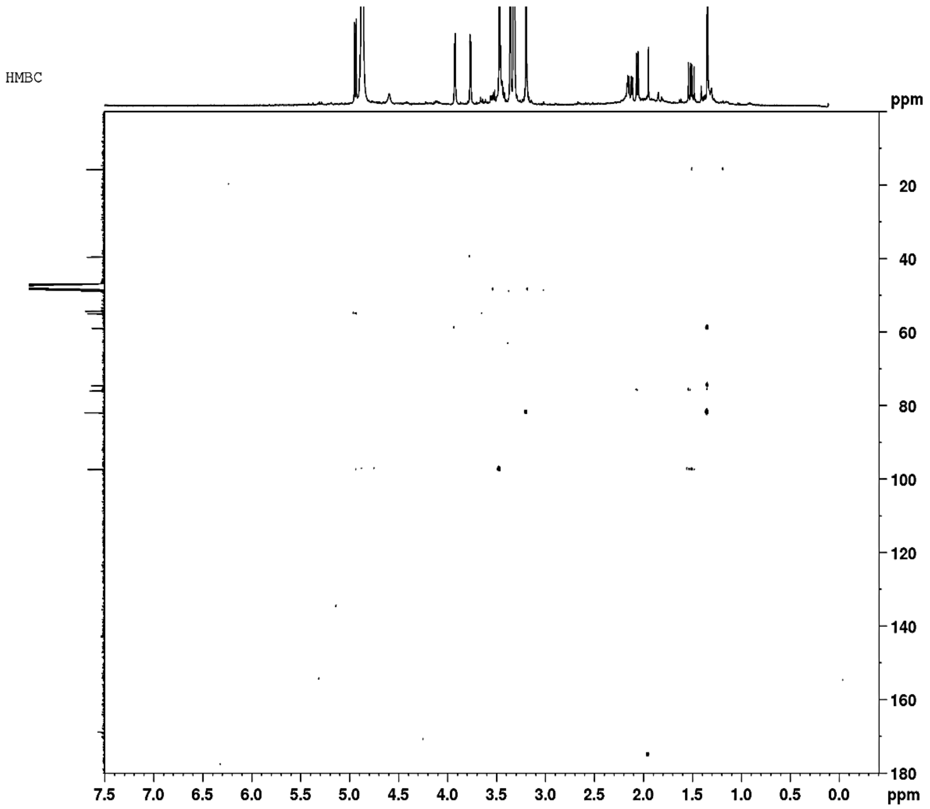

Figure S6. HMBC spectrum of compound **1**

Table S2. ^{1}H-NMR and ^{13}C-NMR assignments for mussaenosidic acid (**2**) recorded in D_2O.

**Position**	**δ_H (ppm), multiplicity, *J* (Hz)**	**δ_C (ppm)**
**1**	5.6; d; *J*= 2.8	94.3
**3**	7.47; s	151.0
**4**		112.6
**5**	3.10; m	29.5
**6**	2.23 (2H, m)	28.7
**7**	1.74 (1H, m):1.52 (1H, m)	39.6
**8**		79.6
**9**	2.36; m	50.6
**10**	1.30; s	22.9
**11**		171.2
**1’**	4.80 (1H, overlapped with solvent)	98.2
**2’**	3.07-3.52; m	72.6
**3’**	3.07-3.52; m	77.2
**4’**	3.07-3.52; m	69.5
**5’**	3.07-3.52; m	76.2
**6a’**	3.92; dd; *J*=2.0, 12.0	60.6
**6b’**	3.72; dd; *J*=6.0, 12.0	

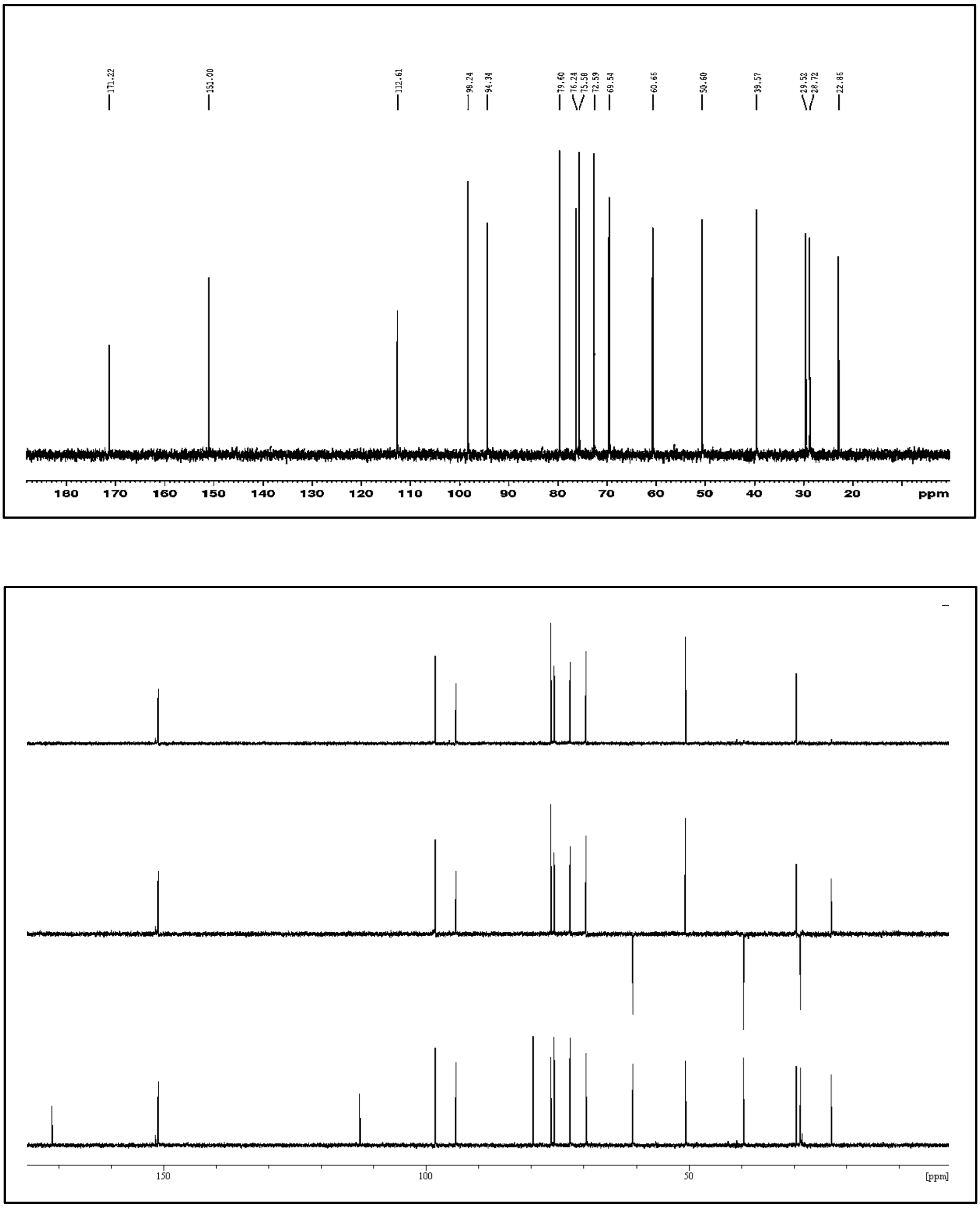

Figure S7. ^{13}C-NMR spectra including DEPT-135 and DEPT-90 of mussaenosidic acid (**2**) in D_2O.

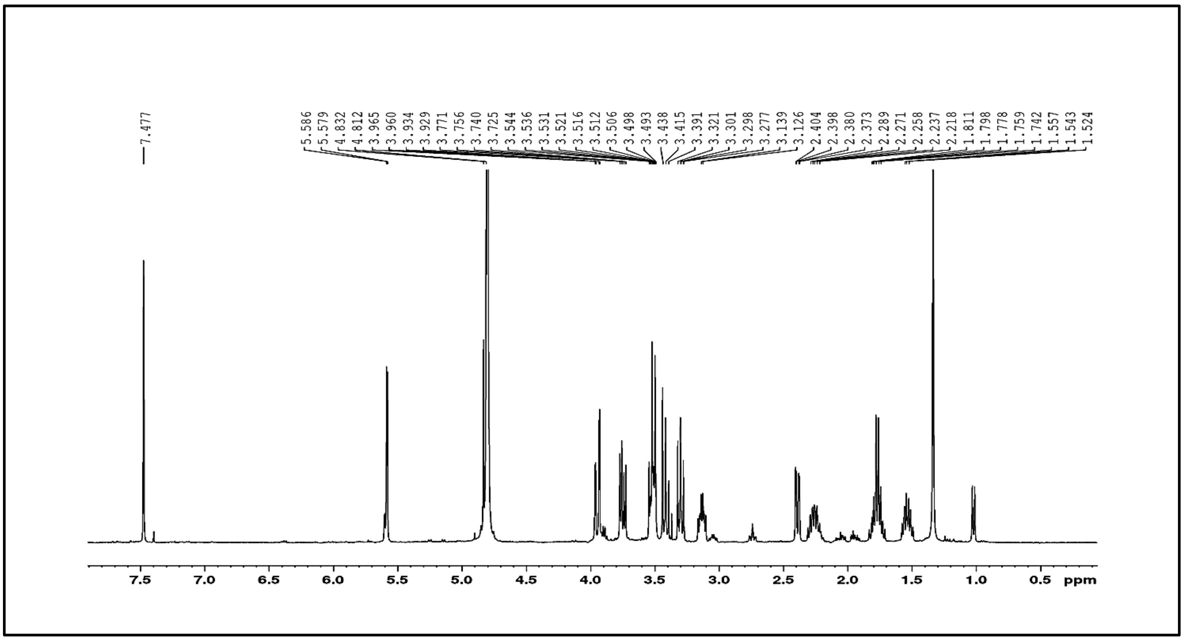

Figure S8. ^{1}H-NMR spectrum of mussaenosidic acid (**3**) in D_2O.

Table S3. ^{1}H-NMR and ^{13}C-NMR assignments for mussaenoside (**3**) recorded in methanol-d_4.

**Position**	**δ_H (ppm); multiplicity; *J* (Hz)**	**δ_C (ppm)**
**1**	5.48; d; *J*=4.2	95.3
**3**	7.4; s	152.0
**4**		113.3
**5**	3.20; m	32.0
**6**	2.30(1H, m) / 1.45(1H, m)	30.7
**7**	1.75 (2H, m)	40.7
**8**		80.5
**9**	2.24; dd; *J*=4.2,9.3	52.3
**10**	1.33; s	24.6
**11**		169.4
**1'**	4.25; d; *J*=7.8	99.8
**2'**	3.0-3.72; m	74.7
**3'**	3.0-3.72; m	78.4
**4'**	3.0-3.72; m	71.7
**5'**	3.0-3.72; m	78.0
**6_a'**	3.92; dd; *J*= 2.0,11.8	62.9
**6'$_b$**	3.65; dd; *J*= 6.2,11.8	
**OMe**	3.71; s	51.6

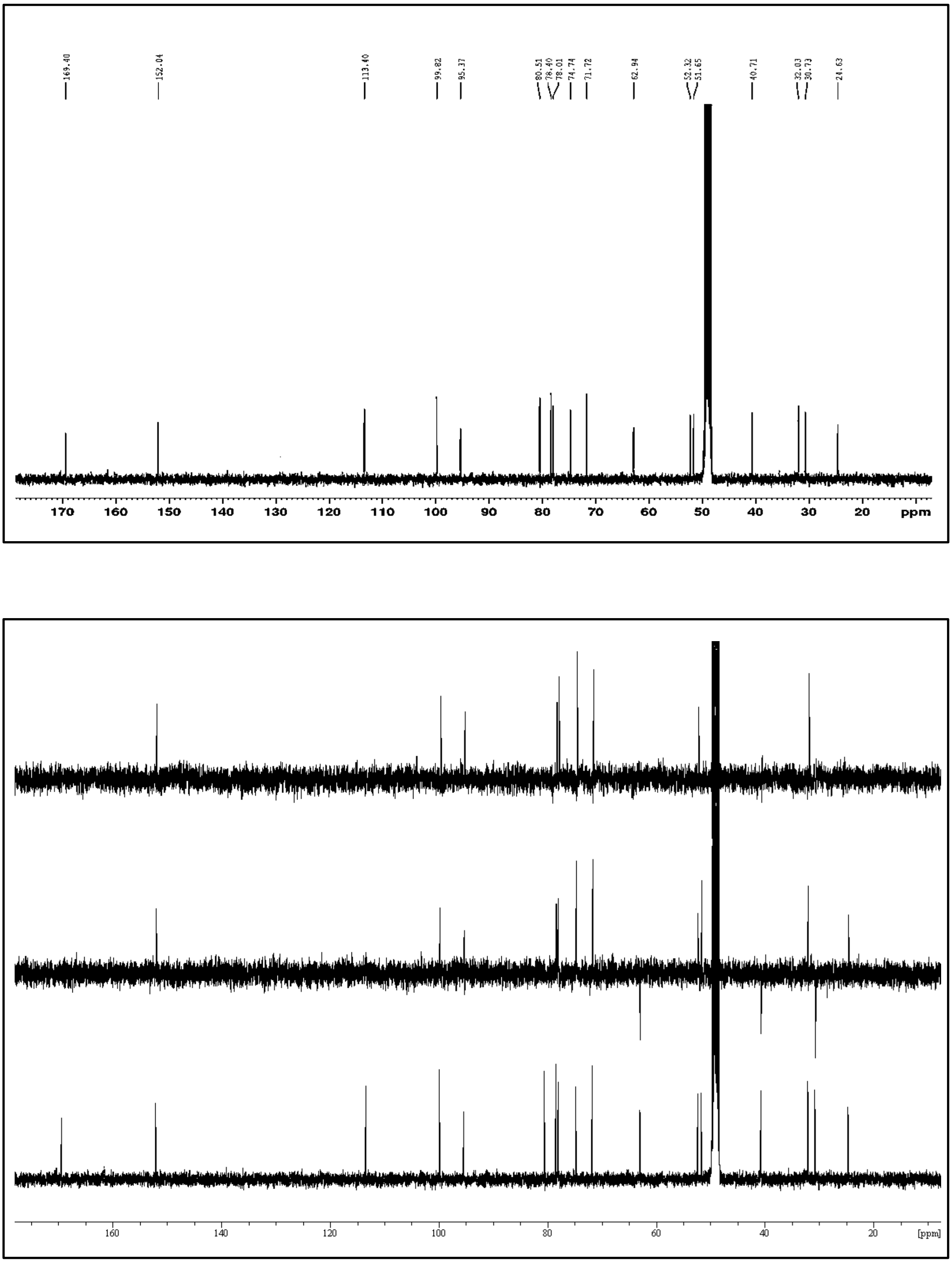

Figure S9. ^{13}C-NMR spectra including DEPT-135 and DEPT-90 of Mussaenoside (**3**) in methanol-d_4.

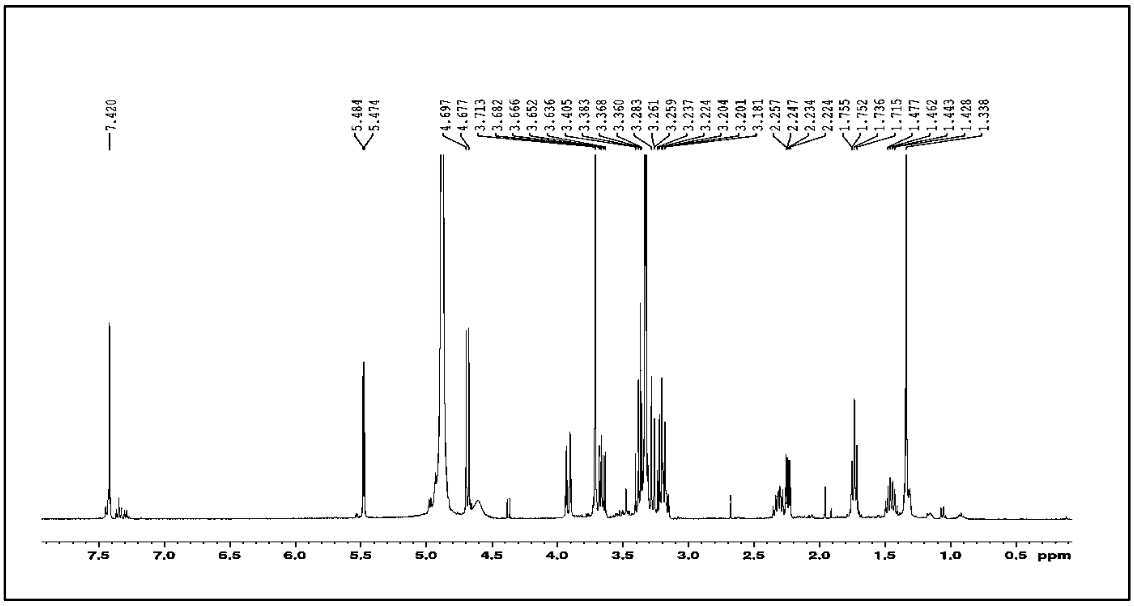

Figure S10. ^{1}H-NMR spectrum of Mussaenoside (**3**) in methanol-d_4.

Table S4. ^{1}H-NMR and ^{13}C-NMR assignments for Linarioside (**4**) recorded in D_2O.

**Position**	**δ_H (ppm); multiplicity; *J* (Hz)**	**δ_C (ppm)**
**1**	5.80; s	91.0
**3**	6.42; d; *J*=6.5	139.2
**4**	5.30; dd; *J*=6.5, 1.2	108.1
**5**		64.6
**6**	3.42-4.2; m	79.2
**7**	3.42-4.2; m	72.5
**8**		74.0
**9**	2.57; s	56.2
**10**	1.30; s	17.1
**1'**	4.20; d; *J*= 7.8	97.8
**2'**	3.42-4.2; m	75.2
**3'**	3.42-4.2; m	79.2
**4'**	3.42-4.2; m	69.7
**5'**	3.42-4.2; m	76.2
**6'**	3.42-4.2; m	61.6

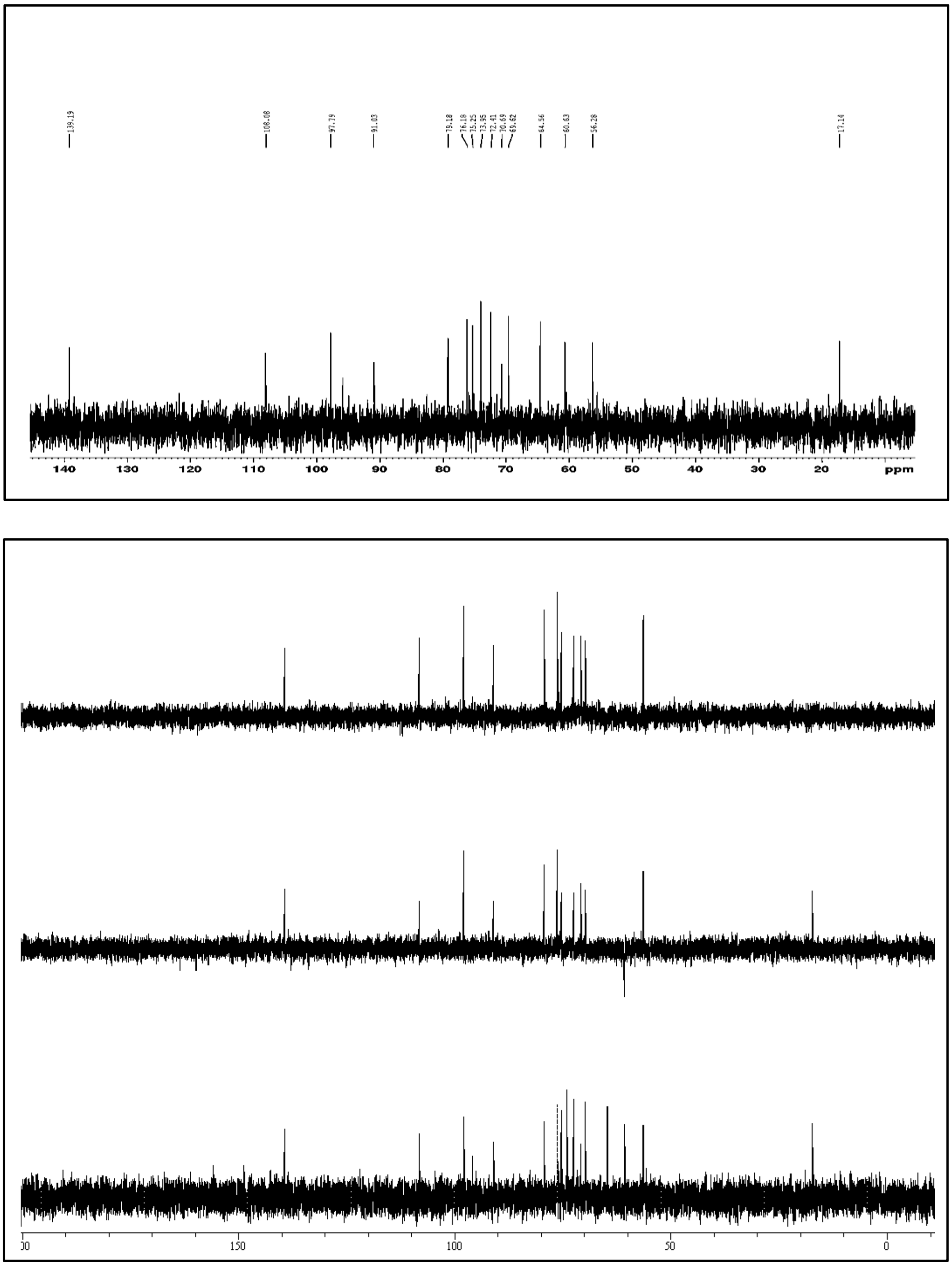

Figure S11. ^{13}C-NMR spectra including DEPT-135 and DEPT-90 of linarioside(**4**) in D_2O.

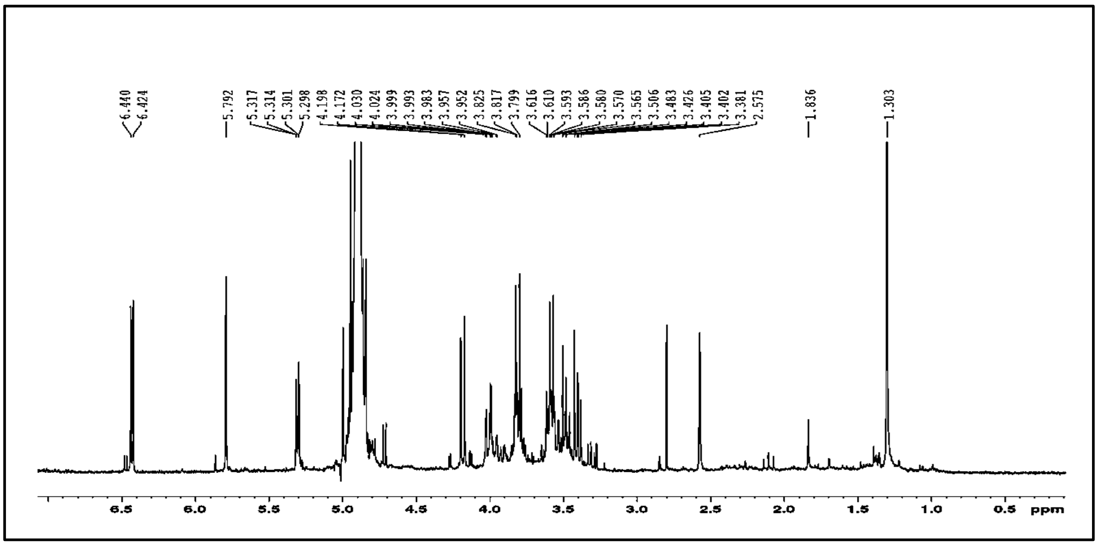

Figure S12. ^{1}H-NMR spectrum of Linarioside (**4**) in D_2O.

Table S5. ^{1}H-NMR and ^{13}C-NMR assignments for pectolinarin (**5**) recorded in $CDCl_3$.

**Position**	**δ_H (ppm), multiplicity, *J* (Hz)**	**δ_C (ppm)**
**1**		
**2**		164.4
**3**	6.53; s	103.9
**4**		183.0
**5**		152.2
**6**		130.4
**7**		155.0
**8**	6.51; s	93.4
**9**		153.1
**10**		105.8
**1’**		123.6
**2’**	7.77; d; *J*=9.0	128.2
**3’**	6.97; d; *J*=9.0	114.5
**4’**		162.8
**5’**	6.97; d; *J*=9.0	114.5
**6’**	7.77; d; *J*=9.0	128.2
**OMe (C-6)**	3.98; s	61.0
**OMe (C-4’)**	3.84; s	55.6

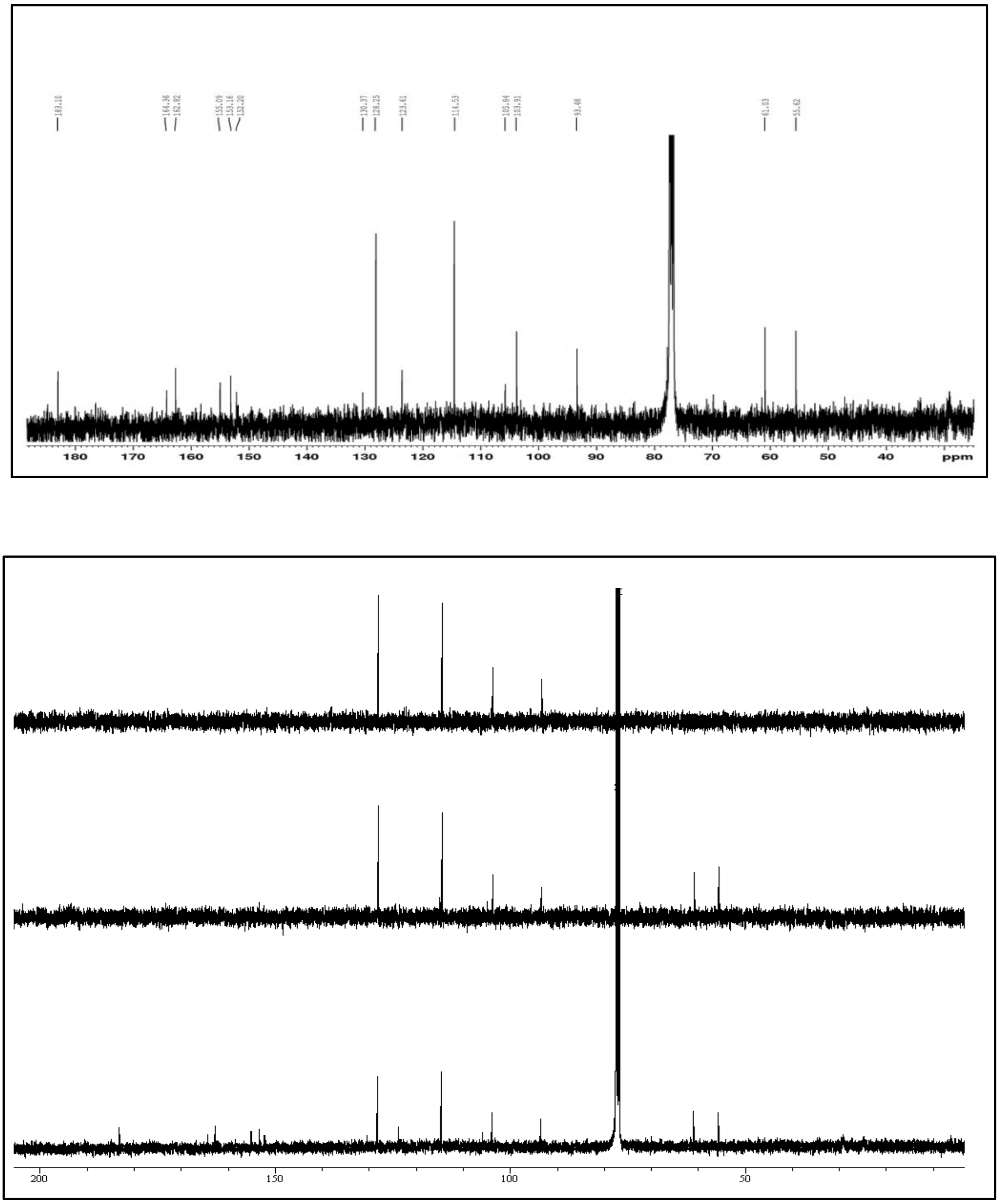

Figure S13. ^{13}C-NMR spectra including DEPT-135 and DEPT-90 of pectolinarigenin (**5**) in $CDCl_3$.

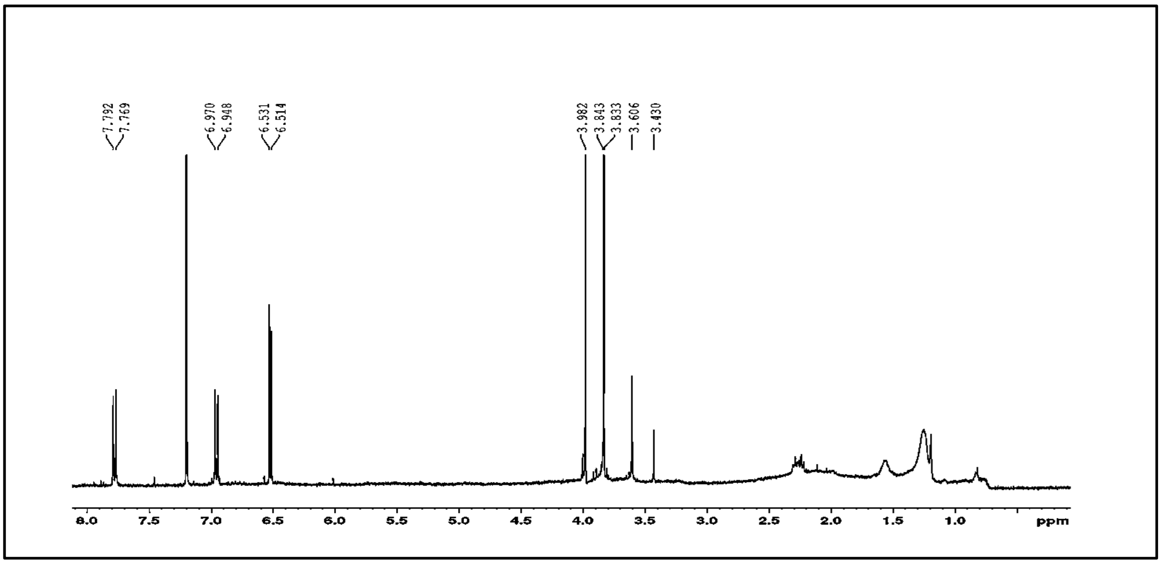

Figure S14. ^{1}H-NMR spectrum of pectolinarigenin (**5**) in $CDCl_3$.

Table S6. ^{1}H-NMR and ^{13}C-NMR assignments for pectolinarin (**6**) recorded in DMSO-d_6.

**Position**	**δ_H (ppm); multiplicity; *J* (Hz)**	**δ_C (ppm)**
**1**		
**2**		164.6
**3**	6.92; s	103.9
**4**		182.8
**5**		152.6
**6**		133.3
**7**		157.0
**8**	6.94; s	94.8
**9**		152.7
**10**		106.3
**1'**		123.2
**2'**	8.04; d; *J*=8.9	129.0
**3'**	7.16; d; *J*= 8.9	115.3
**4'**		163.0
**5'**	7.16; d; *J*= 8.9	115.2
**6'**	8.04; d; *J*=8.9	128.9
**1''**	5.12; d; *J*=7.0	100.9
**2''**	3.12-3.75; m	73.6
**3''**	3.12-3.75; m	76.9
**4''**	3.12-3.75; m	69.9
**5''**	3.12-3.75; m	76.2
**6''**	3.12-3.75; m	66.4
**1'''**	4.56; d;1.1	100.7
**2'''**	3.12-3.75; m	70.9
**3'''**	3.12-3.75; m	71.5
**4'''**	3.12-3.75; m	72.4
**5'''**	3.12-3.75; m	68.2
**6'''**	1.06; d; 6.0	18.2
**OMe(C-6)**	3.76; s	60.8
**OMe(C-4')**	3.85; s	56.0

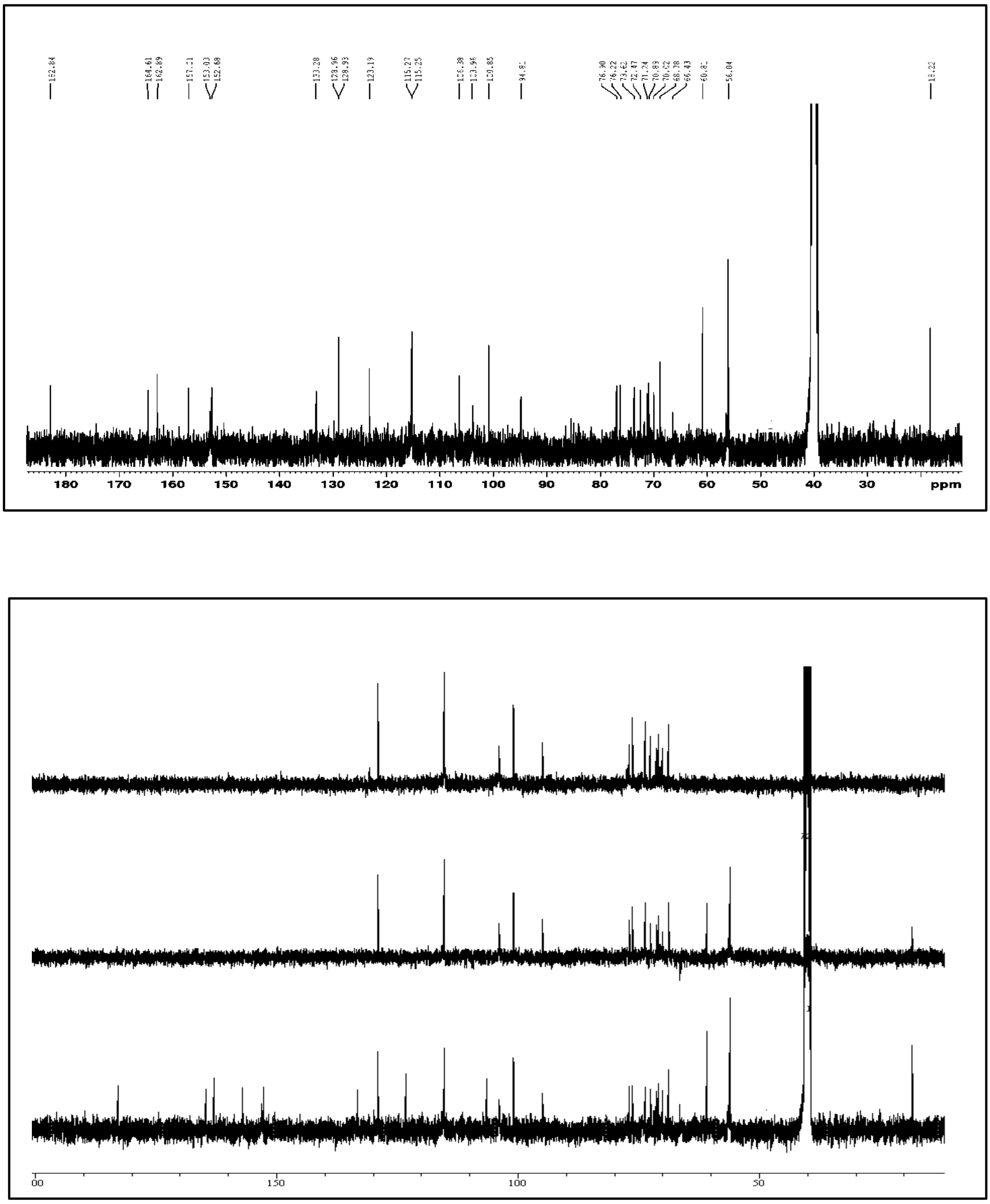

Figure S15. ^{13}C-NMR spectra including DEPT-135 and DEPT-90 of pectolinarin (**6**) in DMSO-d_6.

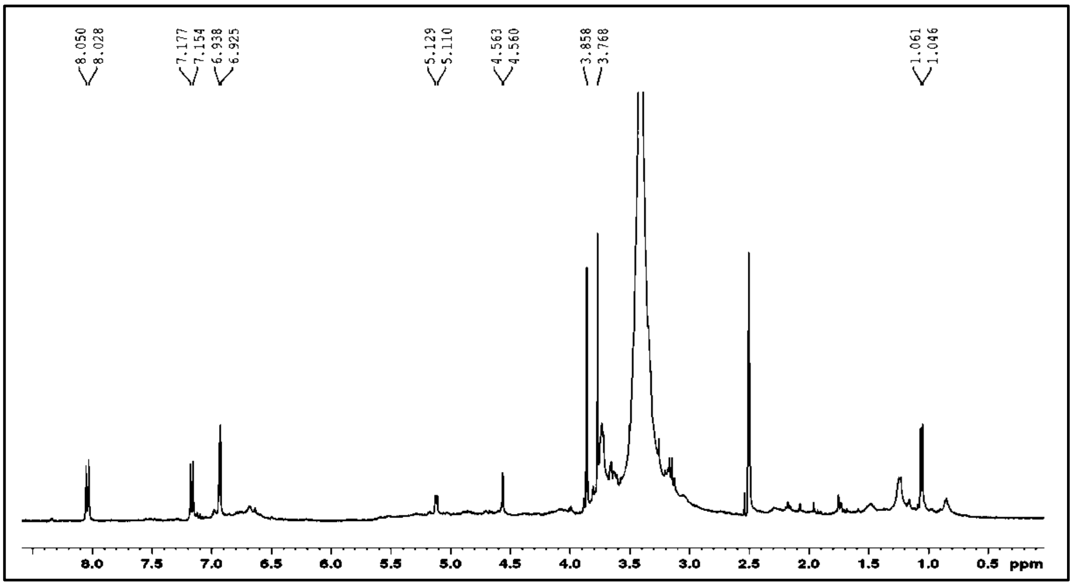

Figure S16. ^{1}H-NMR spectrum of pectolinarin (**6**) in DMSO-d_6.

Table S7. ^{1}H-NMR and ^{13}C-NMR assignments for 4- hydroxy benzoic acid methyl ester (7) recorded in methanol-*d*$_4$.

**Position**	**δ_H (ppm); multiplicity; *J* (Hz)**	**δ_C (ppm)**
**1**		121.8
**2**	7.88; d; *J*= 8.9	132.5
**3**	6.84; d; *J* = 8.9	116.0
**4**		163.6
**5**	6.84; d; *J*= 8.9	116.0
**6**	7.88; d; *J*= 8.9	132.5
**7**		168.6
**OMe**	3.86; s	52.0

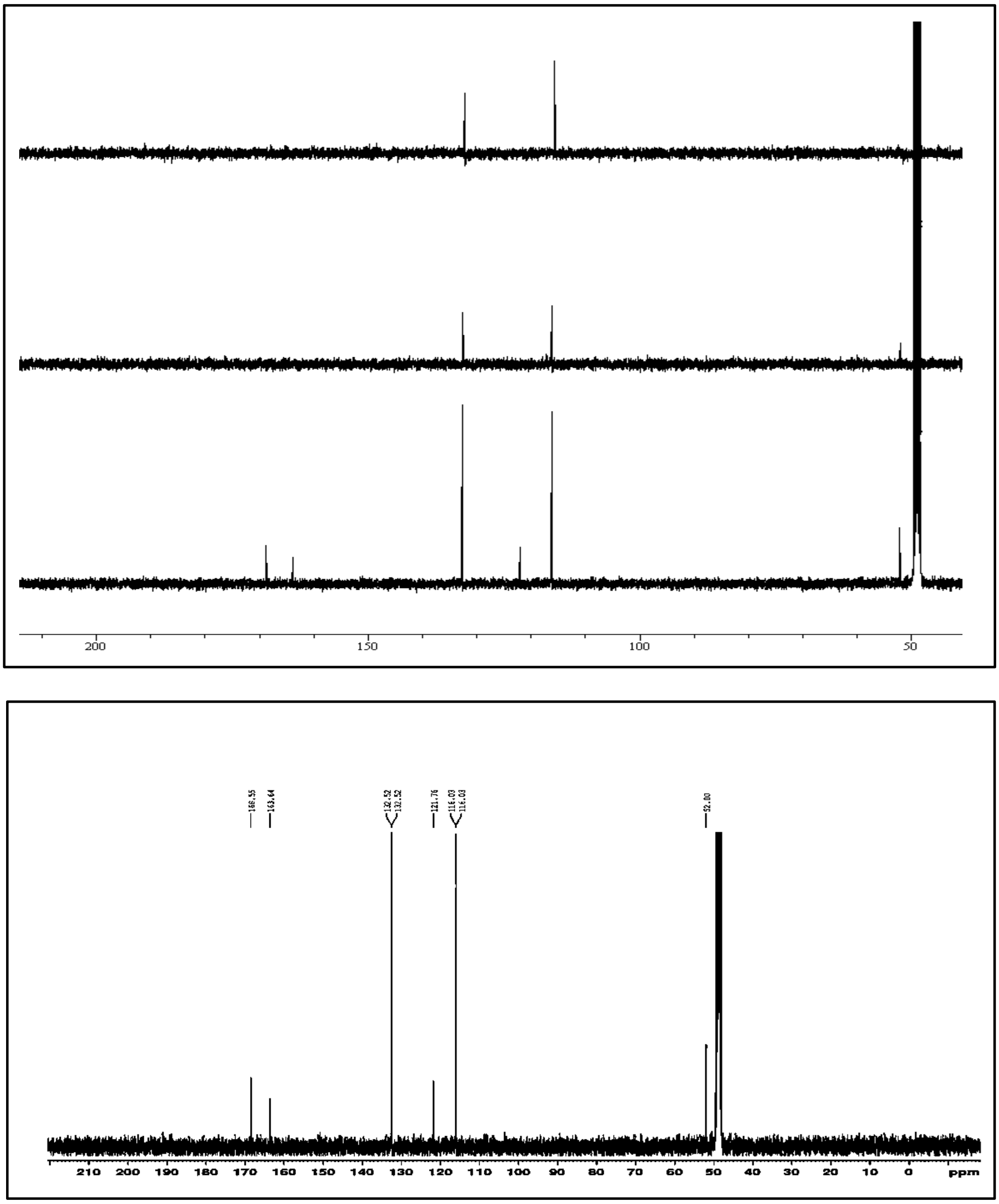

Figure S17. ^{13}C-NMR spectra including DEPT-135 and DEPT-90 of 4-hydroxy-benzoic acid methyl ester (**7**) in methanol-d_4.

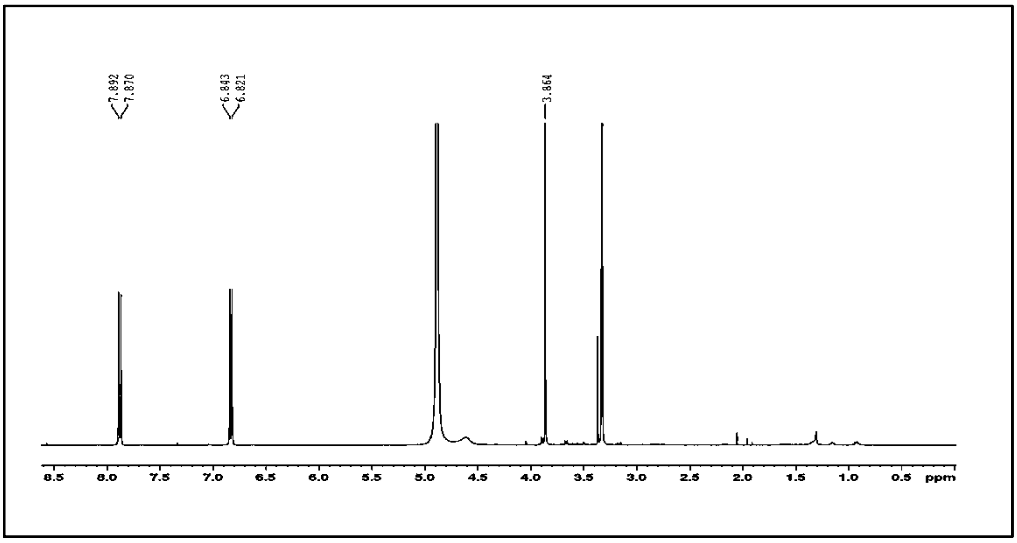

Figure S18. ^{1}H-NMR spectrum of 4-hydroxy-benzoic acid methyl ester (**7**) in methanol-d_4.
